# Selective small-chemical inhibitors of protein arginine methyltransferase 5 with anti-lung cancer activity

**DOI:** 10.1371/journal.pone.0181601

**Published:** 2017-08-14

**Authors:** Gui-Mei Kong, Min Yu, Zhongping Gu, Zhi Chen, Rui-Ming Xu, Deon O'Bryant, Zhengxin Wang

**Affiliations:** 1 Medical School of Yangzhou University, Yangzhou, China; 2 School of Life Sciences, Yunnan University, Yunnan, China; 3 Department of Thoracic Surgery, Tangdu Hospital, Fourth Military Medical University, Xi’an, China; 4 National Laboratory of Biomacromolecules, Institute of Biophysics, Chinese Academy of Science, Beijing, China; 5 Department of Biological Sciences, Clark Atlanta University, Atlanta, GA, United States of America; University of South Alabama Mitchell Cancer Institute, UNITED STATES

## Abstract

Protein arginine methyltransferase 5 (PRMT5) plays critical roles in a wide variety of biological processes, including tumorigenesis. By screening a library of small chemical compounds, we identified eight compounds that selectively inhibit the PRMT5 enzymatic activity, with IC_50_ values ranging from 0.1 to 6 μM. Molecular docking simulation and site-directed mutagenesis indicated that identified compounds target the substrate-binding site in PRMT5. Treatment of lung cancer cells with identified inhibitors led to inhibition of the symmetrical arginine methylation of SmD3 and histones and the cellular proliferation. Oral administration of the inhibitor demonstrated antitumor activity in a lung tumor xenograft model. Thus, identified PRMT5-specific small-molecule inhibitors would help elucidate the biological roles of PRMT5 and serve as lead compounds for future drug development.

## Introduction

Protein arginine methyltransferase (PRMT) enzymes transfer a methyl group from S-adenosylmethionine (AdoMet) to the arginine side-chains in histones and other proteins [1–5]. PRMT enzymes are evolutionarily conserved in eukaryotes and can be divided into types I through IV based on their patterns of arginine methylation [6]. Type I PRMTs modify proteins by the catalysis of asymmetric *N*^*G*^,*N*^*G*^-dimethylarginine, whereas type II enzymes catalyze the formation of symmetric *N*^*G*^,*N*^*G*^-dimethylarginine. Type III PRMTs catalyze the formation of *N*^*G*^-monomethylarginine, and type IV PRMTs modify the delta nitrogen atom of the arginine residue. All PRMTs have a common catalytic (methyltransferase) domain consisting of a highly conserved core region of around 310 amino acid residues and subdomains important for binding to AdoMet and to the substrate [7–10]. The individual PRMT family members differ in unique N-terminal or/and C-terminal regions, which contain distinct domain motifs and perform different functions. PRMTs methylate histones, transcription factors and transcriptional cofactors to regulate gene expression. Many PRMT substrates are associated with RNA; arginine methylation has been implicated in mRNA splicing, transport, and turnover [2]. Studies have also indicated that PRMTs may play important roles in signal transduction as well as protein nucleocytoplasmic shuttling. Because protein arginine methylation is an important posttranslational modification process involved in essential cellular functions, deregulation of PRMTs or their substrates contributes to several different pathogenic processes including those in cancer and cardiovascular disease [4].

Protein arginine methyltransferase (PRMT5), the major type II methyltransferase [11], has been implicated in diverse cellular and biological processes, including transcriptional regulation [4,12,13], RNA metabolism [2,14], ribosome biogenesis [15], Golgi apparatus structural maintenance [16], and cell cycle regulation [12]. In mammalian cells, PRMT5 localizes to both the cytoplasm and the nucleus and methylates multiple histones and nonhistone proteins [2]. In the nucleus, PRMT5 has been found in the SWI/SNF and nucleosome remodeling and deacetylase chromatin-remodeling complexes [17,18], where it can methylate histones as well as transcription factors or regulators [4,12,13] and inhibit cell growth [19]. In the cytoplasm, PRMT5 forms a 20S protein arginine methyltransferase complex, termed “methylosome,” consisting of spliceosomal snRNP Sm proteins, PRMT5, pICln, and WD repeat protein (WDR77/MEP50/WD45/p44) [20–22]. In this complex, PRMT5 methylates Sm proteins [20,23] and such methylation increases the binding affinity of these Sm proteins for the survival motor neuron (SMN), the spinal muscular atrophy disease gene product [24,25]. Subsequently, PRMT5 and SMN complexes cooperate to load the Sm proteins onto U snRNAs, forming U snRNPs [26]. Also in the cytoplasm, PRMT5 is essential for cell proliferation [19,27]. The importance of PRMT5 in cancer is demonstrated by its upregulation in several human malignancies [28] and essential role in growth of lung cancer cells and tumor xenografts [19,27].

Small-molecule inhibitors of PRMTs have been used to elucidate the biological roles of arginine methylation. As an AdoMet analog, sinefungin competes for AdoMet binding and inhibits the activity of all AdoMet-dependent methyltransferases, including PRMTs [29]. Removal of the methyl group from AdoMet generates S-adenosylhomocysteine, which also acts as a methyltransferase inhibitor. Methylthioadenoine has been reported to inhibit methyltransferase activity via AdoMet catabolism; however, these inhibitors and similar molecules are not specific to the PRMT pathways as they inhibit other AdoMet-dependent enzymes. Non-nucleoside small-molecule inhibitors (arginine methylation inhibitors) of PRMTs were identified by Bedford and coworkers by screening a chemical compound library [29]. A few PRMT5-selective inhibitors have been discovered using various screening methods or by creating analogs to arginine methylation inhibitors [30]. These inhibitors selectively inhibited PRMT5 activity as well as growth of some cancer cells or tumor xenografts. We performed an ELISA-based screening method and identified eight PRMT5-selective inhibitors. Identified compounds inhibited growth of lung cancer cells and lung tumor xenografts in the nude mouse.

## Materials and methods

### Human subject research

The study does not involve human subjectives.

### Animal research

Nude mice were used for the study. The Morehouse Medical School Institutional Animal Care and Use Committee approved all of the experimental procedures. Mice were handled in accordance with the guidelines published in the National Institutes of Health Guide for the Care and Use of Laboratory Animals.

### Chemicals

The DIVER^set^ chemical compound library containining10,000 individual compounds from a diverse collection of synthetic chemicals was purchased from ChemBridge Corporation (San Diego, CA). All compounds meet the high quality standard of 100% identification by NMR and/or LC-MS and have a purity level of at least 90%. Eight identified compounds and one analogue compound (100 mg/each compound) were also purchased from ChemBridge Corporation. These nine compounds meet the high quality standard of 100% identification by NMR and LC-MS and have a purity level of at least 95%.

### Protein expression and purification

Human PRMT1, PRMT3, PRMT5, SmD3, and E2F1 cDNAs were subcloned into pET15d (Novagen). These plasmids were transfected into BL21(ED3) cells. His6x-tagged proteins were expressed and purified using Ni-NTA Agarose (QIAGEN) as reported [19]. Histones H2A, H2B, H3, and H4 were purified form HeLa cells as reported elsewhere [31]. PRMT5 and WDR77 were co-expressed in bacteria and purified as a stoichiometric complex of two polypeptides as reported previously by us [19]. The recombinant human histones H4 and H2A were purchased from Sigma-Aldrich.

### Screening for compounds

Ten-thousand small-molecule compounds from a DIVER^Set^ library (ChemBridge) were screened as follows: High-binding 96-well plates (Fisher Scientific) were coated with 100 ng of recombinant SmD3 (the substrate) at 4 ^o^C overnight. After blocking with 5% BSA for 30 min at room-temperature, PRMT5-WDR77 (800 ng) was added to each well. DIVER^Set^ compounds were then transferred to individual wells to a final concentration of 20 μM. The enzyme reaction was initiated by the addition of the methyl donor (AdoMet, 10 μM). Reaction mixtures were incubated for 3 h at 30 ^o^C. The wells were then washed twice with TBST (25 mM Tris [pH 7.5], 150 mM NaCl, 0.1% Tween 20) and blocked in TBST containing 2% BSA for 30 min. The methylarginine-specific primary antibody ab412 (Abcam) (1:1,000) and secondary antibody (anti-mouse IgG-peroxidase at 1:10,000; Fisher Scientific) were added to each well and the mixtures were incubated at room-temperature for 1 h. After three washes with TBST, 0.1 ml of 3,3′,5,5′-tetramethylbenzidine (TMB) substrate (0.1 mg/ml) was added to each well. Wells were incubated for 5 min in the dark at room-temperature and the reaction was stopped by addition of 0.1 ml of 2 M H_2_SO_4_ per well. Potential “hits” were compounds associated with at least a 90% reduction in optical density at 450 nm (in duplicate).

### Radioactive methyltransferase assay

Methylation reactions were performed as previously described with a few modifications [19,31]. Reaction mixtures (40 μl) containing 1.2 μg of PRMT5, 98 ng of PRMT1 or 98 ng of PRMT3; 2 μg of SmD3 or histones; 1 μCi of S-[methyl-^3^H]adenosylmethionine (H^3^-AdoMet; PerkinElmer); and 5 μM AdoMet were incubated in 50 mM Tris-HCl (pH 7.5)–1 mM EGTA–1 mM EDTA at 30°C for 2 h. Reaction mixtures (20 μl) were boiled in 5 μl of 5x SDS sample buffer and separated on a 15% polyacrylamide gel. Gels were fixed for 30 min in 40% methanol–10% acetic acid, incubated in 20 ml of Amplify (Amersham Life Science) for 10 min, dried and exposed to X-ray film at -80°C. IC_50_ was calculated by linear regression analysis of percent inhibition. The other half (20 μl) of each reaction mixture was spread onto DE81 chromatography paper discs (Whatman). The paper discs were washed 4 times with 50 mM NaHCO_3_ and dried. The amounts of methylated products were quantified by liquid scintillation.

### Molecular docking

The program Glide [32,33] was used to dock all of the designed compounds into the binding site of PRMT5 as well as PRMT1. In the docking process, the crystal structure of PRMT5 (PDB code 3UA3) and PRMT1 (PDB code 3Q7E) were used, respectively. The water molecules were deleted. Target protein protonation was performed with the pprep script in Glide. Glide constructed a grid that defined the ligand-binding site search region which was centered on the co-crystallized S-Adenosyl-L-homocysteine (SAH) in the complex structures, respectively. The grid was defined as an enclosed box with similar size of SAH in all three dimensions. For the docking runs, the extra precision (XP) docking mode was selected. The compounds in mol2 format were transferred into mae format with the mol2convert script and then docked flexibly into the binding site. The pose with the best GlideScore for each ligand was saved for further analysis.

### Time-dependent inhibition assay

Different amounts of compound C2 or C5 were present in the final methylation reaction mixtures with 1.2 μg of PRMT5, 5 μM AdoMet, 2 μCi of H^3^-AdoMet and various concentrations of SmD3. After a 10-, 20- or 30-min incubation at 30 ^o^C or no incubation, the reaction mixtures were spread onto DE81 chromatography paper discs and the amounts of methylated products were quantified by liquid scintillation. Amounts (counts per minute [cpm]) of methylated products and incubation time (minutes) were plotted and reaction velocity (cpm per minute) was deduced.

### HPLC chromatography

Waters 2695 HPLC System and a C18 column (Symmetry, 3.6 x 75 mm) were used. The column was equilibrated with 10% acetonitrile and eluated with a linear gradient from 10% acetonitrile to 100% acetonitrile in 60 min. Compounds were dissolved in 10% acetonitrile and the purchased compounds (ChemBridge) were used as the standards.

### Cell culture and the treatment with compounds

Lung cancer A549 (ATCC) and PC14 (ATCC) cells were cultured in minimum essential medium (Cellgro) with 10% (v/v) fetal bovine serum (FBS) (HyClone), 2% vitamins, 1% L-glutamine, 1% non-essential amino acids and 1% sodium pyruvate. For the treatment with identified compounds, cells (2, 000 cells/well) were seeded in 24-well plates in the medium containing 2% FBS. The fresh medium containing identified compounds was added 24 hours later. Cells were cultured for various times in a CO_2_ incubator and the cell numbers were counted. Each experiment was performed in triplicate.

### Histone isolation

Histones were purified as reported previously [34]. Briefly, cells (1–5 x 10^6^) or lung tumors (10–100 mg) were lysated using a glass dounce in 1 ml of 1x Passive Lysis Buffer (Promega) and nuclear pellets were collected by centrifugation at 3,000 rpm for 5 min at 4 ^o^C. The supernatant was discard and the pellets were washed with 1 ml of 1x Passive Lysis Buffer. Nuclear pellets were resuspended in 0.4 ml of the buffer (10 mM Trs-HCl, pH7.9, 0.2 mM EDTA, 1 mM DTT, 20% glycerol, 0.42 M KCl) and incubate for 30 min on ice, and centrifuged at 12,000 rpm for 10 min at 4 ^o^C. The supernatant (histones extract) was transferred to a new tube and histones were precipitated by 20% trichloroacetic acid.

### Western blot analysis

Protein extracts or histones (20 μg) were subjected to 10% or 15% sodium dodecyl sulfate-polyacrylamide gel electrophoresis, respectively then transferred to Immobilon-P membranes (Millipore). The blots were then probed for 2 hours with the primary antibody at dilutions of 1:500 (anti-symmetrical dimethylated arginine, SYM10, EMD Millipore), 1:1,000 (anti-B Raf, ab65050, Abcam) or 1:1,000 (anti-PRMT5, Abcam). The blots were then incubated with a horseradish peroxide-conjugated secondary antibody (1:5,000; Fischer Scientific) for 1.5 h. Immunoreactive proteins were detected using an enhanced chemiluminescence detection system (GE Healthcare) per the manufacturer’s instructions. Protein concentrations were determined using the Bradford protein assay reagent (Bio-Rad).

### Measured serum concentrations of identified compounds

Male BALB/c mice (8–10 weeks old) were purchased from Charles River Laboratory and were randomized for compound administration. Chemical compounds were dissolved in 0.1 ml of 0.5% (w/v) carboxymethycellulose (Sigma-Aldrich) plus 0.5% Tween 80 (Fisher Scientific) and administrated by oral gavage at the dosage of 50 mg/kg or 100 mg/kg. Mice were sacrificed and blood samples were collected immediately before (n = 5) and at 2 (n = 5), 4 (n = 5), 6 (n = 5) or 8 (n = 8) h after compound administration. Plasma samples were sent to the Pharmaceutical Development Center at MD Anderson Cancer Center for LC-MS/MS assay to determine compound concentrations.

### Lung orthotopic tumors and the compound treatment

Six-week old nude mice were purchased from the National Cancer Institute and maintained in a barred animal facility. Cell injection into the mouse lung was performed as described previously [35]. Mice were treated with vehicle [0.1 ml, 0.5% (w/v) carboxymethycellulose plus 0.5% Tween 80] by oral gavage or the compound dissolved in the vehicle (0.1 ml, 100 mg/kg) for 21 days. The lungs of the mice were then removed, fixed with formaldehyde and embedded in paraffin. Paraffin-embedded lung tissue sections (4 μm) were stained with H&E and evaluated for tumors. The tumor areas in lungs were quantified using the ImageJ software program (NIH).

### Statistical analysis

Data are presented as the means of three independent experiments ± the standard deviation. A 2-tailed unpaired Student *t*-test was used to determine whether differences between control and experimented samples were statistically significant. *, *p*<0.05; **, *p*<0.01; ***, *p*<0.001. *P* values less than 0.05 were considered statistically significant.

## Results

### Screening for PRMT5 inhibitors

**Fig 1:**
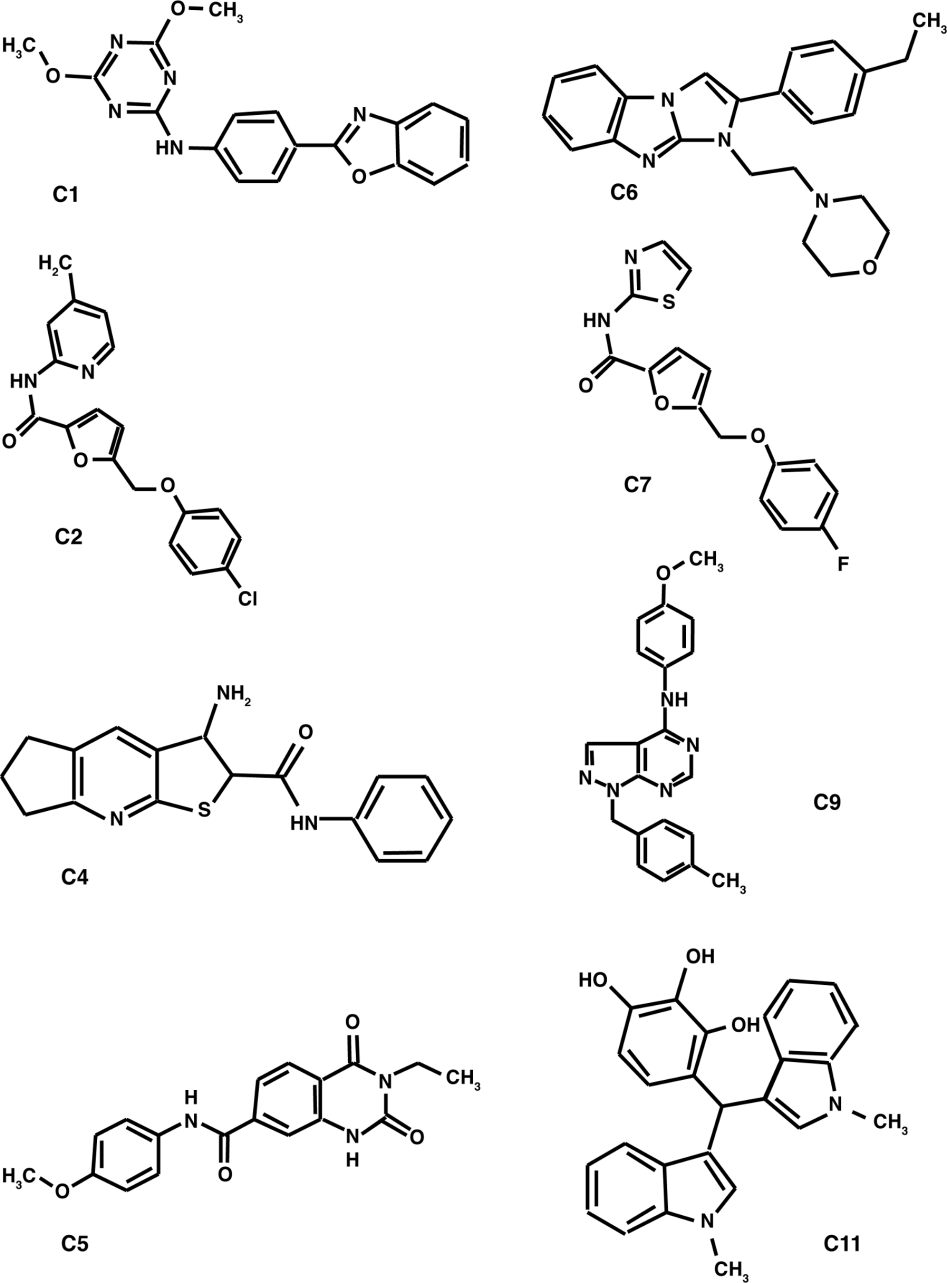
Chemical structures of identified chemical compounds.

Cellular spliceosome protein SmD3 was methylated by PRMT5 *in vitro* and *in vivo* [23]. Mouse monoclonal antibody (ab412 [Abcam]) reacts with *N*^*G*^-monomethyl and *N*^*G*^*-N*^*G*^-dimethyl arginine and does not recognize unmodified arginine. This antibody was used to establish an enzyme-linked immunosorbent assay (ELISA)-based screen for small molecule inhibitors of arginine methylation of SmD3 by PRMT5. The recombinant SmD3 was immobilized on a 96-well plate and incubated with the human recombinant PRMT5-WDR77 complex. Small molecules were added to individual wells using a 12-channel pipette. The methylation reaction was initiated by addition of the methyl donor (AdoMet). The arginine-methylated SmD3 was detected by the monoclonal antibody ab412 and a peroxidase-conjugated secondary antibody. The screen was performed on 10,000 individual compounds from a diverse collection of synthetic chemicals (DIVER^set^, ChemBridge). We identified eight compounds that dramatically (>90% at the concentration of 20 μM) inhibited methylation of SmD3 by PRMT5 (Fig 1, Table 1). Compounds C2 and C4 are structurally related to compounds C7 and C6, respectively. These compounds are not structurally related to the known PRMT5 inhibitors [30]. The IC_50_ (concentrations required to inhibit enzyme activity by 50%) values of the compounds ranged from 0.1 to 6 μM (Table 1). These compounds inhibited arginine methylation activity of PRMT5 similarly to the PRMT5-WDR77 complex. We selected five compounds (C2, C5, C6, C9, and C11) for further analysis.

**Table 1:** List of some properties of identified compounds.

Sample ID	ChemBridge ID	Formular	Molecular Weight	LogSW	tPSA	IC50
C1	7554223	C18H15N5O3	349	-6.36	95.2	0.50
C2	7561729	C18H15CIN2O3	343	-5.26	64.4	0.10
C4	6244219	C17H15N3OS	309	-4.89	68.0	0.60
C5	7661491	C18H17N3O4	339	-3.62	93.2	0.75
C6	5790435	C23H26N4O	447	-5.93	34.7	0.50
C7	7494331	C15H11FN2O3S	318	-4.56	64.4	2.5
C9	7664243	C20H19N5O	345	-5.45	64.9	6.0
C11	7630081	C25H22N2O3	398	-7.83	70.6	0.30
C9a	9032604	C18H16N6	316	-4.31	81.6	0.70

### Identified compounds selectively inhibited PRMT5

**Fig 2:**
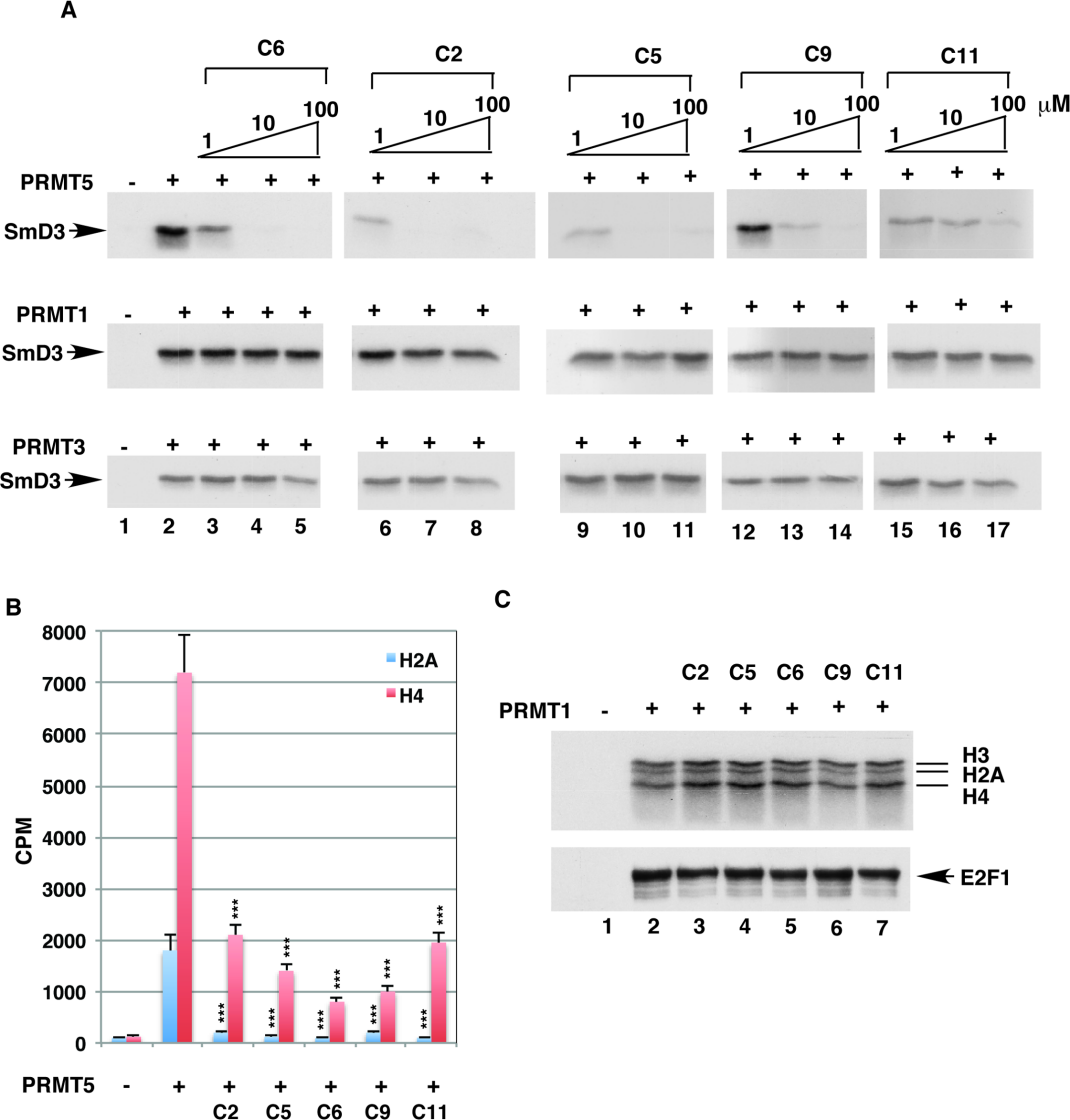
Identified compounds selectively inhibited the methyltransferase activity of PRMT5. In vitro methylation reactions were performed with recombinant PRMT5 (1.2 μg), PRMT1 (89 ng) or PRMT3 (89 ng); [H^3^]-AdoMet; and different substrates in the presence of identified compounds. A, Identified compounds inhibited methylation of SmD3 by PRMT5. The recombinant SmD3 (2 μg) was used as the substrate in the absence (lane 2) or presence of 1, 10 or 100 μM compounds as indicated. Reaction mixtures were separated on an SDS–15% polyacrylamide gel and the gel was dried under vacuum and exposed to film. Lane 1 shows the product of the reaction without the enzyme. B, Identified compounds inhibited methylation of histones H2A and H4 by PRMT5. Recombinant histones H4 or H2A (1 μg) were used as substrates of PRMT5 in the methylation assay in the absence or presence of 100 μM compounds as indicated. Reaction mixtures were spread onto DE81 chromatography paper discs. After a wash, the amounts of methylated products on discs were quantified by liquid scintillation. C, Identified compounds did not inhibit methylation of histones and E2F1 by PRMT1. PRMT1 was incubated with purified histones (1 μg) or recombinant E2F1 (1 μg) as substrates in the absence (lane 2) or presence of 100 μM compounds as indicated. Reaction mixtures were separated on an SDS–15% polyacrylamide gel and the gel was dried and exposed to film.

To determine whether the five compounds inhibit PRMT5 selectively, we examined their inhibition of other PRMT isoforms (type I PRMTs), PRMT1 and PRMT3, using the radioactive methyltransferase assay. The results are shown in Fig 2. The recombinant PRMT1 and PRMT3 were active when assayed against the SmD3 substrate (Fig 2A, lane 2). In contrast to dramatic PRMT5 inhibition, none of the five compounds significantly inhibited SmD3 methylation by PRMT1 or PRMT3 at concentrations up to 100 μM (Fig 2A, lanes 3–17). Similarly, the five compounds inhibited PRMT5 methylation of recombinant histones H2A and H4 (Fig 2B) but not PRMT1 methylation of histones (H2A, H3, and H4) or E2F1 (Fig 2C). Thus, the five compounds have high specificity for PRMT5 inhibition.

### Binding model analysis of inhibitor-enzyme interaction

**Fig 3:**
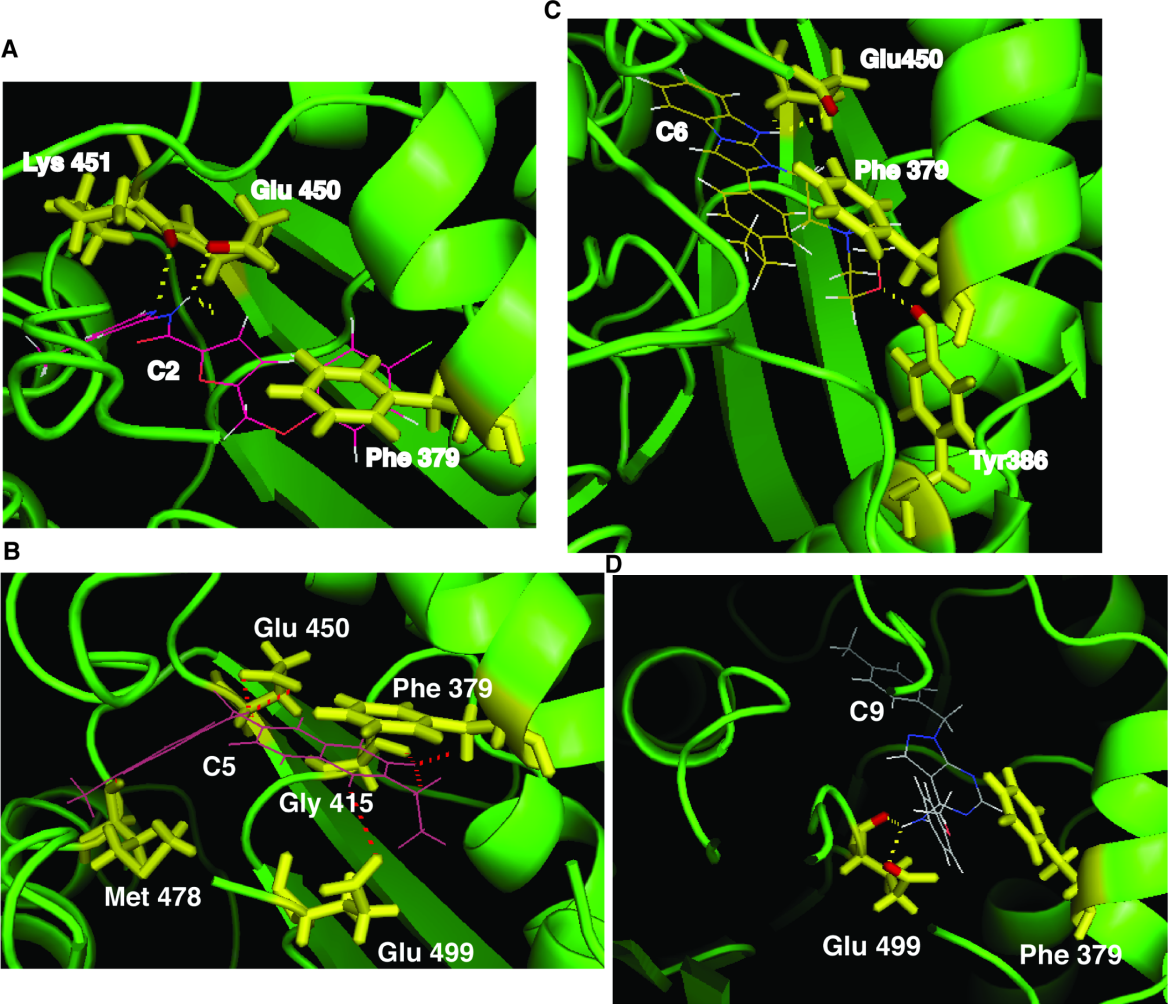
The compounds interact with the substrate-binding region in PRMT5. Docking of compounds C2, C5, C6 or C9 in PRMT5. The amino acid residues in PRMT5 likely to interact with compound C2, C5, C6 or C9 are indicated in yellow.

To understand the structural basis of the identified compounds’ inhibition of PRMT5, we used the program Glide to investigate the docking of the compounds to PRMT5. Each compound was individually docked in *Caenorhabditis elegans* PRMT5 [10]. Docking results were analyzed according to the lowest energy order. The docked ligands were found to distribute only in the substrate-binding region and none of them overlapped with other regions, including the AdoMet-binding site. For example, compound C2 interacts with the Glu450 and Lys451 residues in PRMT5 (Fig 3A) and compound C5 interacts with the Gly415, Glu450, Met478 and Glu499 residues (Fig 3B). The compound C6 interacts with the Glu450 and Tyr386 residues (Fig 3C). Compound C9 interacts with the Glu499 residue (Fig 3D). The conserved Phe379 in the active site of PRMT5 is critical for symmetric addition of methyl groups and changing the residue to methionine (Met) converted PRMT5 to an enzyme that catalyzes both symmetric and asymmetric dimethylation of arginine [10]. We noticed that the compounds seem to have an aromatic-aromatic interaction (aromatic stacking/π stacking) with Phe379. The aromatic ring of compound C2, C5, C6, or C9 and the aromatic side chain of Phe379 are seen in an off-center, parallel orientation (Fig 3), a position preferred for forming the noncovalent interaction of aromatic stacking [36].

**Fig 4:**
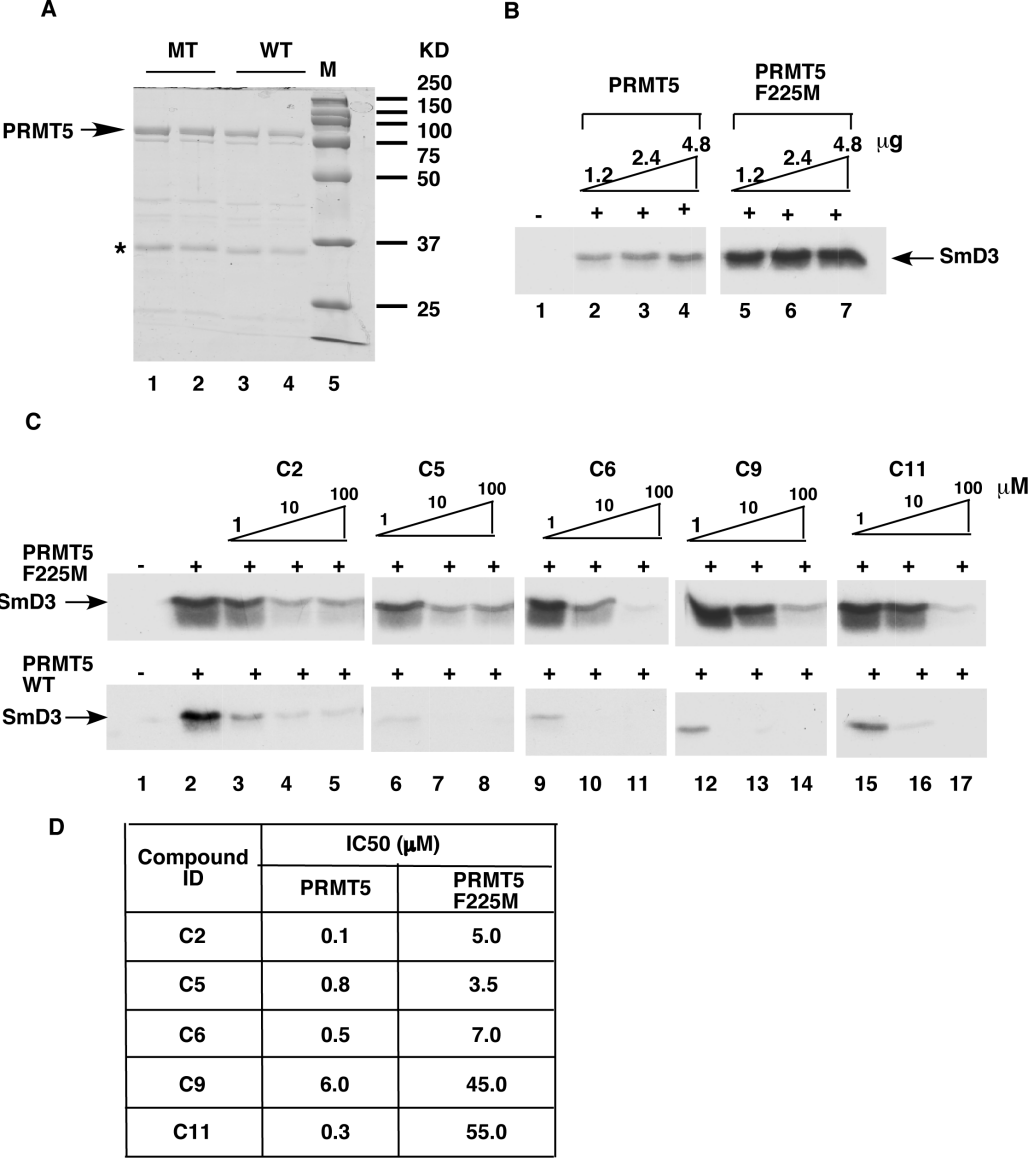
The conserved phenylalanine residue in PRMT5 is critical for inhibition by the five compounds. A, DS-PAGE of recombinant wild type (WT) and F225M mutant (MT) PRMT5. The gel was stained with Coomassie Brilliant Blue R250. A band corresponding to an unknown bacterial protein is indicated (star). B, The F225M mutation increased the methytransferase activity of PRMT5. *In vitro* methylation reactions were performed with recombinant wild type (lanes 2–4) or mutant (lanes 5–7) PRMT5 (1.2, 2.4 or 4.8 μg) and SmD3 (2 μg) as the substrate. C, The F225M mutation in PRMT5 decreased inhibition of arginine methylation by the compounds. The reaction mixture contained SmD3 (2 μg); wild type or mutant PRMT5 (1.2 μg); and 1, 10 or 100 μM compounds as indicated. D, The F225M mutation in PRMT5 decreased the IC_50_ values of the compounds, as determined by quantitation and analysis of methylation assay results.

The inhibitory activity of the compounds may stem from this interaction. To test this possibility, we conducted site-directed mutagenesis to mutate Phe225 in human PRMT5 (corresponding to Phe379 in the *C*. *elegans* PRMT5) to Met. The mutated PRMT5 was expressed in bacteria and purified by Ni-nitrilotriacetic acid (Ni-NTA) affinity chromatography (Fig 4A). The activity of the mutant PRMT5 was examined by the radioactive methyltransferase assay. In agreement with a previous report [10], the mutation of Phe225 to Met significantly enhanced the methylation activity of PRMT5 (Fig 4B). Mutation greatly decreased the ability of the five compounds to inhibit the methyltransferase activity of PRMT5 (Fig 4C). The IC_50_ values for the mutant PRMT5 increased from 4.3 to 183 folds higher than those for the wild type PRMT5 (Fig 4D). These results suggest that the Phe residue interacts with the compounds and that mutation of this residue decreases compound binding and inhibition capacity. The results also can help explain the specificity of the compounds to PRMT5. However, the contribution of this interaction to inhibition varies among the compounds. For example, compound C2 interacts with three amino acid residues (Lys451, Glu450, and Phe379) (Fig 3A) with the mutation of Phe to Met resulting in a 50-times higher IC_50_ (Fig 4C and 4D). In contrast, compound C5 interacts with five amino acid residues (Gly415, Glu450, Met478, Glu499, and Phe379) and mutation of Phe to Met increased IC_50_ only 4.3 times (Fig 4C and 4D), indicating that Phe379 is less important for PRMT5 inhibition by C5.

### Competitive inhibition

**Fig 5:**
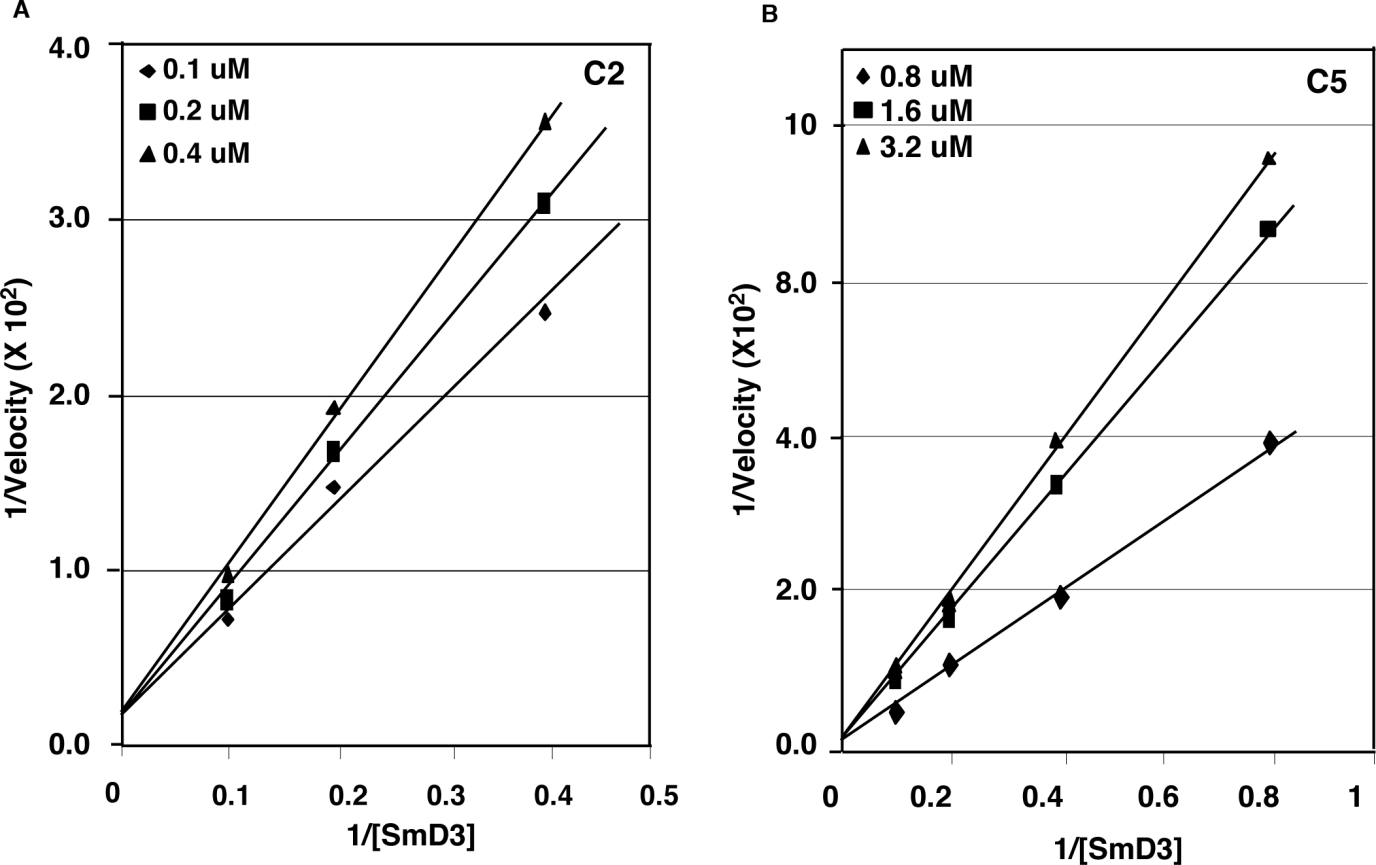
Steady-state kinetic analysis of PRMT5 inhibition by compounds C2 and C5. **Double-reciprocal plot of reaction velocities (cpm per minute) versus concentrations of SmD3.** Methylation reaction mixtures contained various concentrations of C2 (left) or C5 (right), various concentrations of SmD3, 1.2 μg of PRMT5 and 5 μM AdoMet. The mixtures were incubated for different times and the amounts of methylated products were quantified using DE81 paper and liquid scintillation. Reaction velocity was calculated as H^3^ incorporation (cpm) into SmD3 per minute.

To understand the mechanism of inhibition of identified compounds, we analyzed the kinetic pattern of inhibition using steady-state enzymatic measurements. The velocities of PRMT5 methylation of SmD3 were measured at several selected concentrations of compounds C2 and C5 over a range of SmD3 concentrations. Plotted in the double-reciprocal format with 1/velocity versus 1/[SmD3] (Fig 5), the data demonstrate that compounds C2 and C5 are competitive inhibitors with regard to the SmD3 substrate. The similar analysis revealed that compounds C2 and C5 are not competitive inhibitors with regard to SAM. These results agree with the binding model analysis showing that the compounds interact with the active site binding to the protein substrate (Fig 3).

### Identified compounds are cell-permeable and inhibit PRMT5 in lung cancer cells

**Fig 6:**
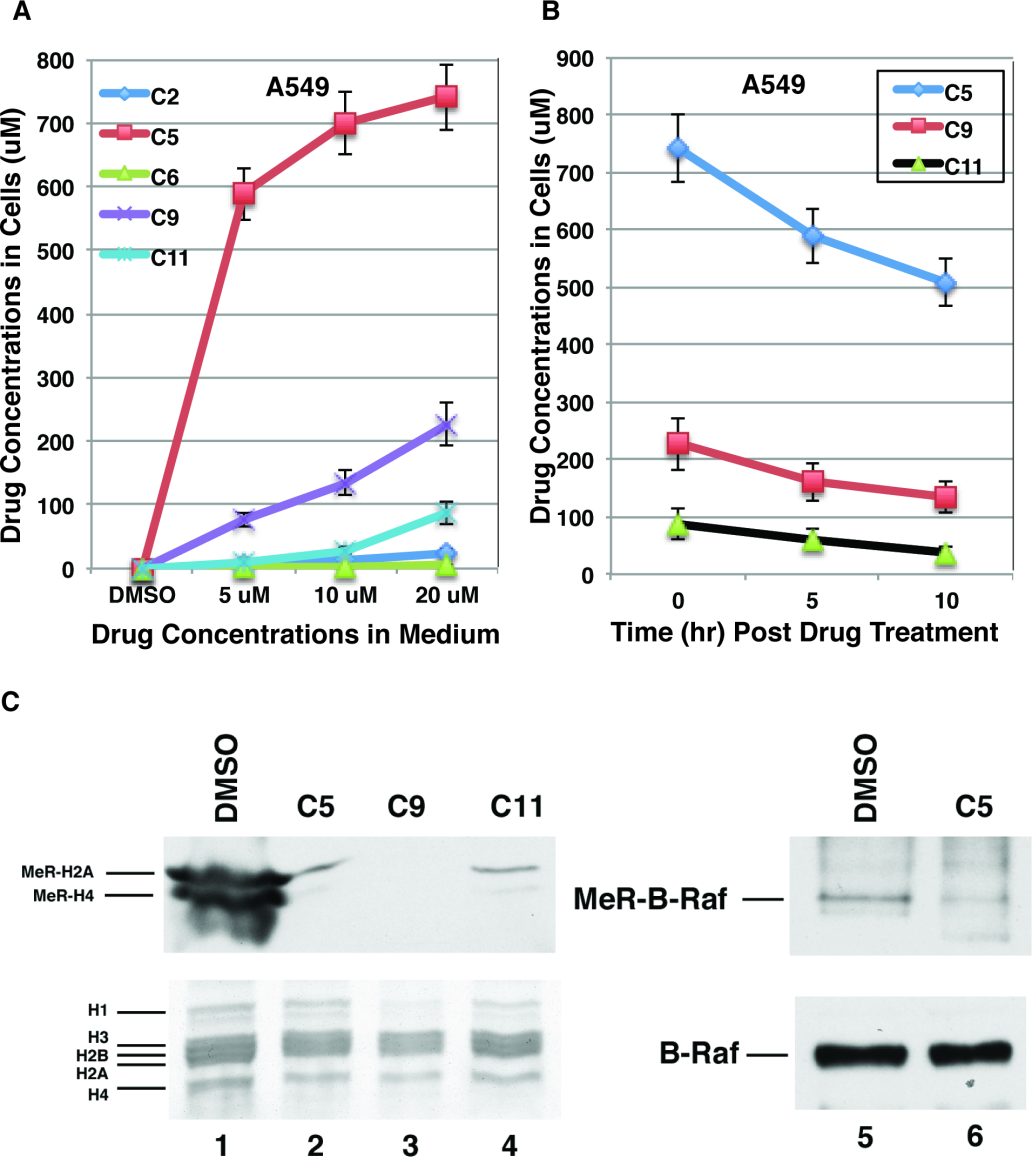
Identified compounds inhibited cellular targets of PRMT5. **A, The cellular permeability of identified compounds.** Lung cancer A549 cells were cultured in the presence of various concentrations of compounds for 2h and the compound concentrations in the cell were determined by HPLC chromatography with a C18 column. B, The cellular stability of identified compounds. Lung cancer A549 cells were cultured in the presence of compounds (20 μM) for 2 h and then the medium was removed. Cells were washed with the medium and cultured for additional 5 or 10 h after the compounds were removed. The compound concentrations in the cell were determined by HPLC chromatography with a C18 column. C, Identified compounds inhibited arginine methylation of histones and B-Raf in A549 cells. A549 cells were grown in the presence of DMSO or compound C5, C9 or C11 (20 μM). Histones were purified and analyzed by SDS-PAGE stained with Commassie blue (lanes 1–4, bottom) or by Western blot with anti-symmetric dimethyl arginine antibody (SYM10) (lanes 1–4, top). B-Raf was immunoprecipitated with anti-B-Raf antibody from whole cell lysates and submitted for Western blot analysis with anti-B-Raf (lanes 5 and 6, bottom) or anti-symmetric dimethyl arginine (lanes 5 and 6, top) antibody.

We then determined whether the identified compounds are cell-permeable. Lung cancer A549 cells were cultured in medium containing various concentrations of the respective compound for 2 hr. Cells were harvested and washed with PBS. The compounds were extracted with ethyl acetate from cells and analyzed by HPLC with a C18 column using the purchased compound as the standard. Compounds C2, C5, C9 and C11 are cell-permeable (Fig 6A), which is consistent with their tPSAs <140 angstroms square (Table 1). In contrast, C6 is not cell-permeable (Fig 6A). The concentrations of compounds C5, C9 and C11 inside cells were even higher than those in the medium (Fig 6A) indicating these compounds were accumulated inside of the cells. We even noticed that the compound C5 crystalized inside the cells. The concentrations of C5, C9 and C11 inside the cells decreased by about 2 folds within 10 h (Fig 6B), indicating that these compounds are relatively stable inside the cells.

To test whether these compounds inhibit PRMT5 in cells, we chose three known substrates (H2A, H4, and B-Raf) of PRMT5 [4,37] to analyze. A549 cells were cultured in the presence of DMSO or three compounds (C5, C9 and C11). Histones and B-Raf were purified and submitted for Western blot analysis with the antibody against the symmetrical dimethyl arginine (SYM10, Millipore) to detect the status of their methylation. Consistent with published observations [4,37], arginine methylation in histone H2A, histone H4 and B-Raf was detected (Fig 6C, lanes 1 and 5) which was significantly inhibited by the compound C5, C9, or C11 (lanes 2–4, 6). Thus, these compounds inhibit symmetrical arginine dimethylation of proteins in cells.

### Identified compounds inhibit growth of lung cancer cells

**Fig 7:**
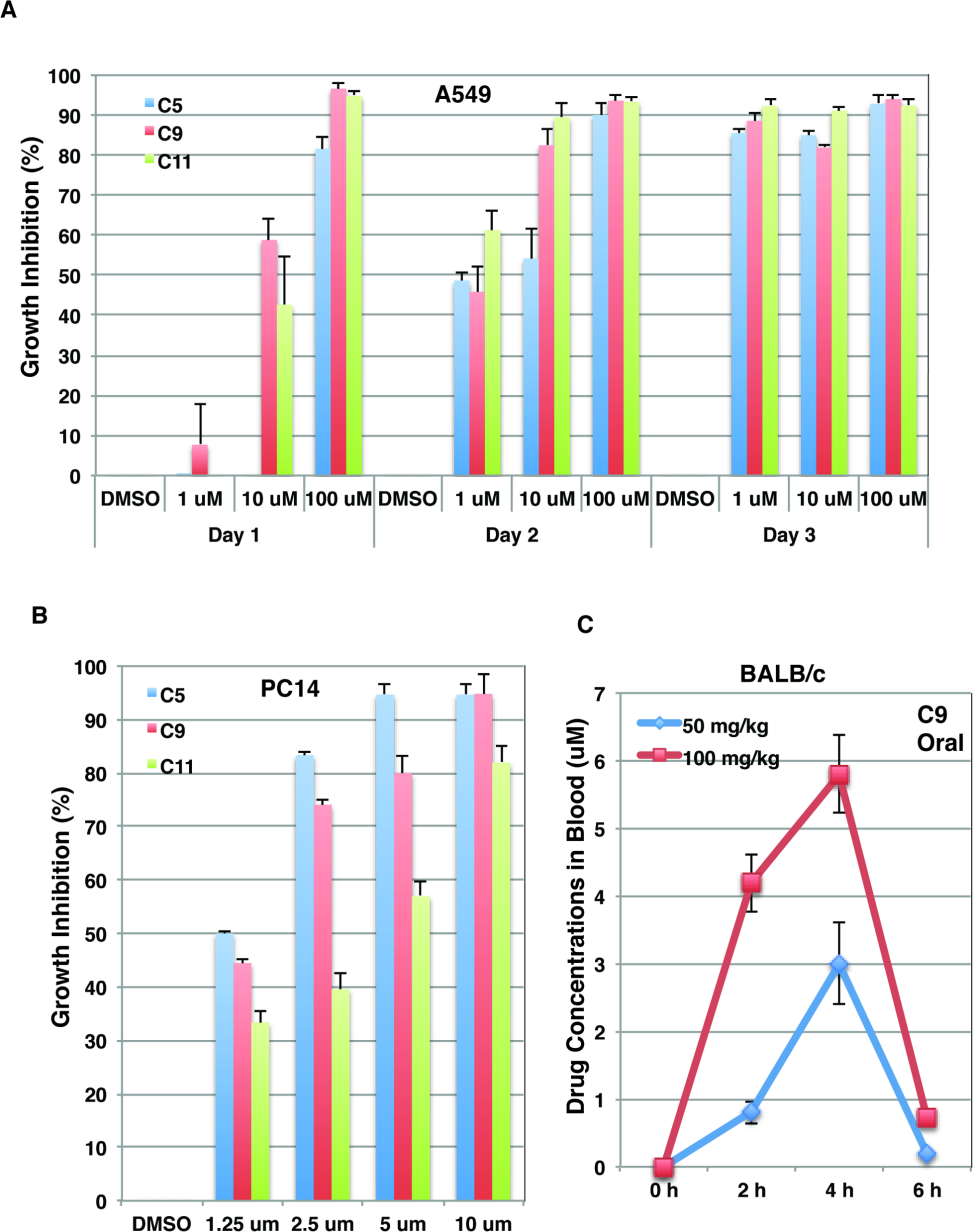
Identified compounds inhibited growth of lung cancer cells. A, Lung cancer A549 cells were cultured in the presence of various concentrations (μM) of identified compounds and growth inhibition was determined by cell counting at various time points. B, Lung cancer PC14 cells were cultured in the presence of DMSO or various concentrations of identified compounds and growth inhibition was determined by cell counting 2 days post the compound treatment. C, The serum concentrations of compound C9 after oral administration. Mice were sacrificed and blood samples were collected immediately before (0 h) and at 2, 4, 6 and 8h after compound administration.

It has been demonstrated that PRMT5 and its enzymatic activity are required for growth of lung cancer cells [19,27]. To test whether identified compounds (C5, C9 and C11) inhibit cell growth, we cultured lung cancer (A549 and PC14) cells in the presence of DMSO or various concentrations of identified compounds. All three compounds inhibited A549 cell growth in a dosage- and time-dependent manner at sub μM concentrations (Fig 7A). Similarly, all three compounds also inhibited PC14 cell growth (Fig 7B). Silencing PRMT5 expression inhibited growth of lung cancer cells by suppressing cellular proliferation and arrested the cell cycle at the G1 phase [27]. Similarly, identified compounds also arrested the cell cycle at the G1 phase (S2 Fig) and inhibited proliferation of lung cancer cells (S3 Fig).

### Oral bioavailability of identified compound

The obtained 3 compounds (C5, C9, and C11) have characteristics of Lipinski’s rule-of-five, which defines four physicochemical parameters found in a set of active drugs which made them into phase II clinical trials [38]. We chose one compound (C9) to analyze its pharmacokinetic parameters in the mouse. BALB/c mice were randomized and administrated compound C9 (dissolved in 5% DMSO and 0.5% Tween 80) at dosages of 25, 50 and 100 mg/kg by intraperitoneal (I.P.) injection. Mice were sacrificed and blood samples were collected at different time points after administration of compounds. The compound concentrations in plasma samples were measured (S1 Fig). The results show that the compound was absorbed quickly into the bloodstream and reached high plasma concentrations (38 μM at the dosage of 100 mg/kg). However, we observed the high toxicity of the compound in the mouse at the dosage of 100 mg/kg.

The oral route of administration is central for delivery of a large number of important drugs in various therapeutic areas and is the most preferred dosage form for cancer treatment [39]. We then administrated the compound C9 by oral gavage formulated in 0.5% (w/v) carboxymethycellulose plus 0.5% Tween 80. Fig 7D shows the time course of compound plasma concentrations following oral administration. The maximal plasma concentration achieved at the dosage of 100 mg/kg is 5.8 μM.

**Fig 8:**
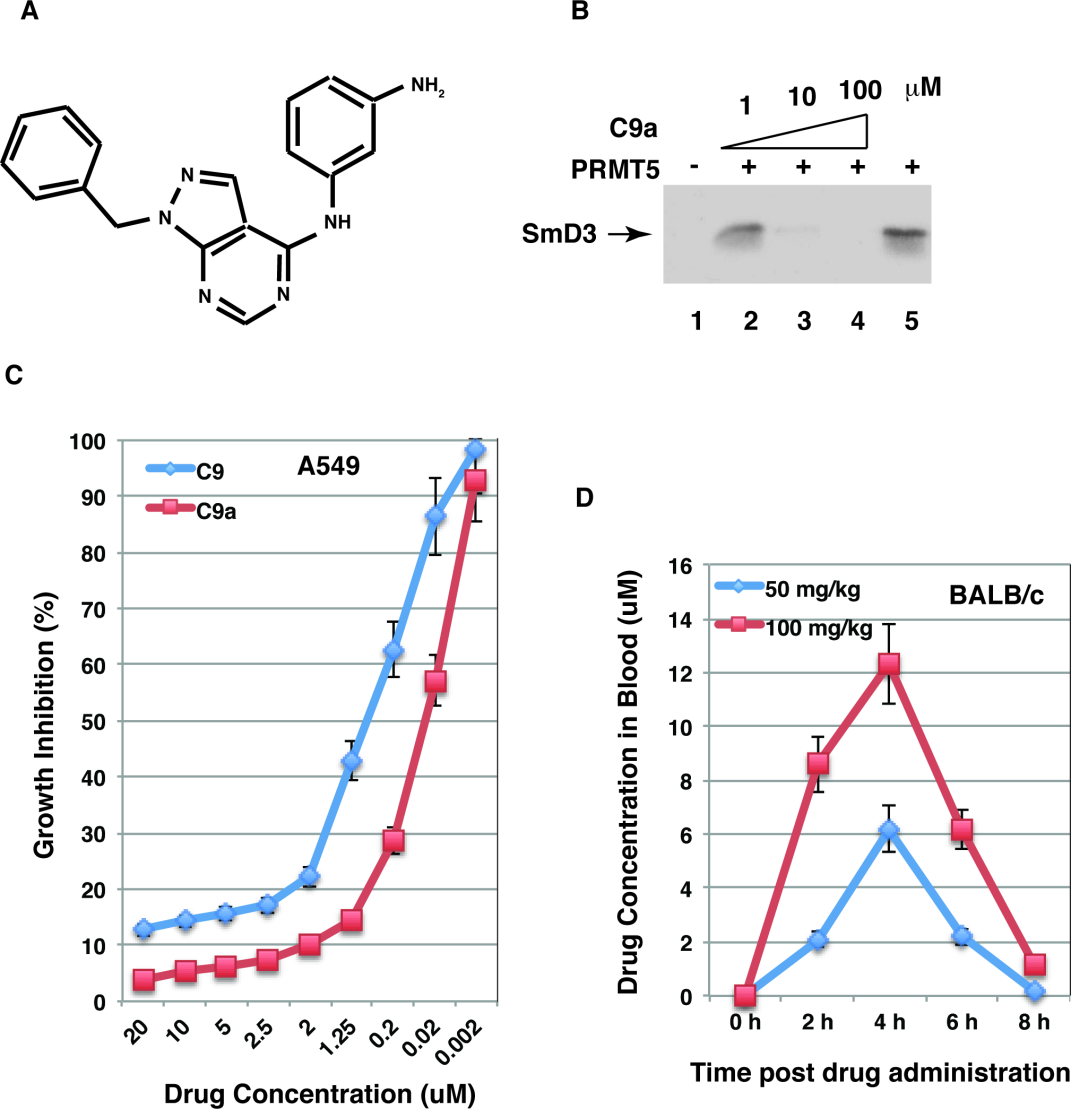
The C9 analogue has improved PRMT5-inhibitory efficacy and oral drug availability. A, The chemical structure of the C9 analogue. B, Compound C9a inhibited methylation of SmD3 by PRMT5. The recombinant SmD3 (2 μg) was used as the substrate in the absence (lane 5) or presence of 1, 10 or 100 μM compounds as indicated. Lane 1 shows the product of the reaction without the enzyme. C, C9a has improved efficacy to inhibit lung cancer growth. A549 cells were cultured in the presence of various concentrations of C9 or C9a and growth inhibition was determined by cell counting 2 days post the compound treatment. D, C9a has improved oral drug availability in the mouse. Male BALB/c mice were randomized and administrated C9a by oral gavage and compound concentrations in blood samples were determined.

About 40% of compounds identified through high-throughput or combinatorial screening approaches are poorly water-soluble [40–42]. Physicochemical properties (such as solubility and permeability) are essential features for oral absorption of drug candidates [42,43]. The poor solubility of drug candidates results in low oral bioavailability and low plasma concentrations [40,44,45]. The low aqueous solubility (LogSW: -5.45) of C9 (Table 1) may lead to low plasma concentrations. The docking result (Fig 3D) indicates that methyl (CH_3_-) and methoxy (CH_3_O-) groups in C9 are not involved in the interaction with PRMT5. By searching the compound library (www.hit2lead.com/search.asp) we found a compound without the methyl (CH3-) group and with an amino group (-NH_2_) substituting the methoxy (CH_3_O-) group of C9 (Fig 8A). The compound has higher (12.6-fold) aqueous solubility than C9 increase (Table 1). As expected, the C9 analogue (C9a) inhibited PRMT5 activity and growth of lung cancer cells with higher efficacy and has improved oral drug availability compared to C9 (Fig 8B, 8C and 8D). The IC_50_ (~1 μM) in the cell growth assay is lower than that (~ 6 μM) in the methylation assay. This may reflect the fact that compound C9 is accumulated (by 5-fold) inside cells (Fig 6A).

### Compound C9a suppressed growth of lung tumor xenografts

**Fig 9:**
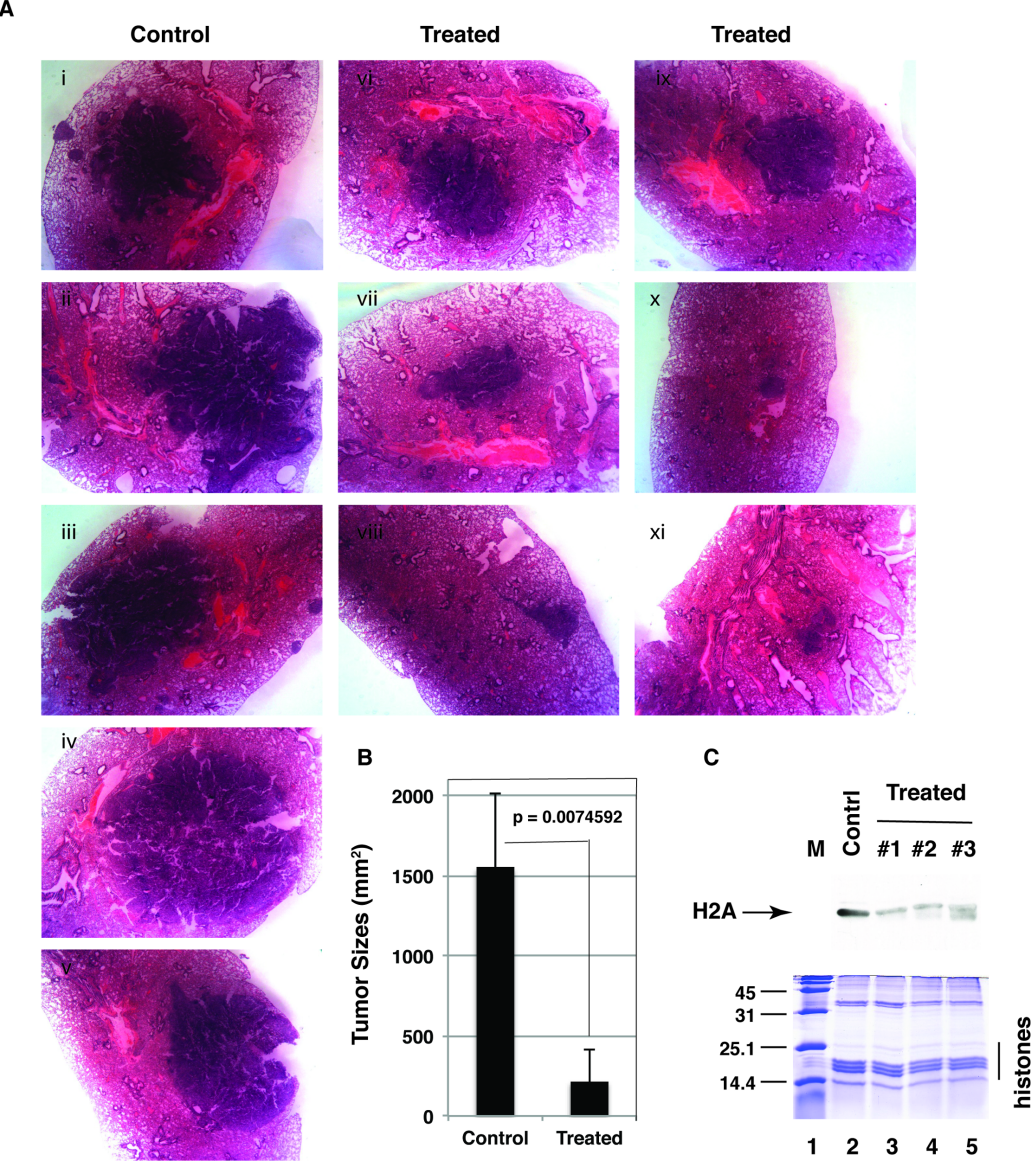
The compound C9a inhibited growth of lung tumor xenografts in nude mice. A, Lungs derived from mice injected with A549 cells treated with the vehicle or C9a at the dosage of 100 mg/kg for 21 days. B, Mean size of tumors. C, The C9a treatment inhibited histone 2A (H2A) arginine methylation in lung tumor xenografts. Histones were isolated from tumors and analyzed by SDS-PAGE stained with Commassie blue R250 (bottom) or by Western blot with anti-symmetric dimethyl arginine antibody (SYM10) (top).

We used the orthotopic lung tumor model to determine effects of the compound C9a on growth of lung cancer xenografts. A549 cells were injected into the lung of nude mouse as previously reported by us [27]. Mice were randomized and divided into two groups: (a) control mice (n = 5): administration of the vehicle [0.5% (w/v) carboxymethycellulose plus 0.5% Tween 80] by oral gavage daily; (b) treated mice (n = 10): administration of the compound C9a in the vehicle at the dosage of 100 mg/kg by oral gavage daily. Mice were treated with the compound C9a for 3 weeks after cell injection. The treatment retarded growth of mice (S4 Fig). Large, macroscopically visible tumors were found in the lungs of five control mice (Fig 9A, i—v). However, tumor sizes in the lungs of the treated mice significantly decreased (Fig 9A, vi—xi). The average tumor sizes (tumor area mean) in the control mice were 7.2-fold bigger than that of the lung tumors in mice treated with the compound C9a (Fig 9B).

Histones were purified from lung tumors and submitted for Western blot analysis with the SYM10 antibody to detect the status of the symmetric dimethylarginine of histones. The arginine methylation in histone H2A was significantly inhibited by the treatment (Fig 9C, lanes 3–5 versus lane 2). Thus, corresponding decreased levels of the symmetrical arginine dimethylated PRMT5 substrate in tumors strongly suggested that the compound C9a 's effect on lung tumors was a direct consequence of PRMT5 inhibition.

## Discussion

In the present study, we identified eight small chemical compounds that specifically inhibit PRMT5. Identified compounds interact with the substrate-binding site of PRMT5 through the π-π interaction with the unique Phe residue in PRMT5. This interaction contributes to the selectivity of identified compounds against other PRMTs. Identified compounds were cell-permeable and inhibited growth of lung cancer cells, which was correlated well with inhibition of symmetric dimerthylation of arginine-containing substrates. Oral administration of one compound demonstrated antitumor activity in the lung tumor xenograft model, with 86% tumor growth inhibition observed after 21 days of the treatment.

In this study, an ELISA-based methylation assay was used to screen a library of 10,000 small chemical compounds. By this approach, we identified eight novel chemical compounds that inhibit PRMT5 but not PRMT1 and PRMT3. More recently, a few studies reported small chemical inhibitors specifically targeting PRMT5. Compounds CMP5 and HLCL-61 specifically inhibited PRMT5 rather than PRMT1, CARM1, or PRMT7 [46,47]. The dual PRMT5-PRMT7 inhibitor (DS-437) is an AdoMet competitor and targets the cofactor-binding site in PRMT5 with an IC_50_ value of 6 μM [48]. From several rounds of screening against a diversity library with 370,000 small molecules and structural optimization, the PRMT5-specific inhibitor EPZ015666 was obtained [49]. EPZ015666 shows outstanding selectivity against the other PRMTs and promising potency (IC_50_ = 0.022 μM). This compound interacts with many of the residues in PRMT5 that involved in peptide binding and form a π-π stacking interaction with Phe327 [49,50]. The inhibitors reported here do not share the structural similarity with these previously identified PRMT5-inhibitors. Although identified compounds are structurally diversified they all target the same site in PRMT5 mainly through the π-π interaction with the unique Phe379 residue in PRMT5.

We identified a set of genes whose expression was altered by silencing PRMT5 expression in lung cancer A549 cells [51]. A549 cells were treated with C9a and EPZ015666 and expression of 6 identified PRMT5-regulated genes was analyzed. Inhibition of PRMT5 enzymatic activity only affeted expression of genes whose expression was suppressed by PRMT5 but no effect on expression of genes whose expression was up-regulated by PRMT5 (S5 Fig). This result suggested that the PRMT5 enzymatic activity may be only required to supress but not to activat gene expression. The future study would use genome-wide approach to determine genes whose expression is affected by PRMT5 inhibitors.

PRMT5 has been shown to be upregulated in a number of different cancers and plays an essential role in growth of various cancer cells [11,28]. Consistent with these observations, PRMT5-inhibitors suppressed growth of lymphoma, AML, and MCL cells in tissue culture [46,47,49]. More recently, EPZ015666 demonstrated antitumor activity in multiple MCL xenograft models [49]. Previous studies used RNA interference technologies revealed an essential role PRMT5 in growth of lung cancer cells and lung tumor xenografts [27]. We have shown in this report that identified PRMT5-inhibitors inhibited growth of lung cancer cells in tissue culture and oral administration of compound C9a suppressed growth of lung tumor xenografts. We noticed that compounds C9 and C9a contain a known kinase inhibitor motif (aminopyrimidine), which exists in some inhibitors of cyclin-dependent kinases (CDKs) (S6A Fig shows chemical structures of the CDK2/5 inhibitor, Roscovitine and CDK4/6 inhibitor, Polbociclib). The retinoblastoma protein (Rb) is phosphorylated by CKDs in cells [52]. Rb is primarily phosphorylated by CDK2 at the site T821 and by CDK4/6 at the site S811, which is inhibited by Roscovitine and Palbociclib, respectively (S6B Fig, lanes 4 and 5). However, compounds C9 and C9a failed to inhibit such phosphorylation events in A549 cells (S6B Fig, lanes 2 and 3), suggesting that compounds C9 and C9a inhibit growth of lung cancer cells not through targeting CDKs rather than a direct consequence of PRMT5 inhibition.

Collectively, identified compounds are powerful probes that could be used to understand more about the biologic roles of PRMT5 and potentially assist in defining a therapeutic strategy for lung cancer treatment.

## Supporting information

S1 Fig The serum concentrations of compound C9. The chemical compound was dissolved in 5% DMSO-0.5% Tween 80. BALB/c mice (8–10 weeks old) were randomized and administrated the vehicle (0.1 ml) or compound C9 dissolved in the vehicle (0.1 ml) by intraperitoneal (I.P.) injection at the dosage of 25, 50 or 100 mg/kg. Mice were sacrificed and blood samples were collected immediately before (0 h, n = 5) and at 2 (n = 5), 4 (n = 5), 6 (n = 5) and 8 (n = 5) h after compound administration. Plasma samples were sent to the Pharmaceutical Development Center at MD Anderson Cancer Center for LC-MS/MS assay to determine compound concentrations.
(TIF)

S2 Fig Identified compounds arrested cell cycle at the G1 phase. A549 cells were infected with the lentivirus expressing non-target (NT) or PRMT5 shRNA as described previously by us [27]. A549 cells were grown in the presence of DMSO or 20 μM of the compound C5, C9 or C11 for 2 days. A, PRMT5 protein levels in A549 cells. Whole cell lysates were prepared and submitted for Western blot analysis with anti-PRMT5 (top) or anti-actin (bottom) antibody. B, Cell cycle distributions. Cells were harvested, washed with phosphate-buffered saline (PBS) and fixed in 70% ethanol at 4 ^o^C overnight. Cells were collected and stained with propidium iodide (PI). The cell-cycle distributions were determined by flow cytometry analysis (BD Accuri^TM^ C6 Flow Cyometer).
(TIF)

S3 Fig Identified compounds inhibited cellular proliferation. A549 cells were infected with the lentivirus expressing NT or PRMT5 shRNA. A549 cells were grown in the presence of DMSO or compound C5, C9 or C11 (20 μM) for 2 days. A, Bromodeoxyuridine (BrdU) (BD Biosciences) incorporation assay. Cells were plated on a Chamber slide (BD falcon) and cultured in the presence of 10 μM BrdU for 4 h. The BrdU-positive cells (brown) were detected by immunostaining with the monoclonal anti-BrdU antibody (BD Biosciences) and observed under a microscope. B, Percentage of BrdU-positive cells in control, PRMT5-silencing or compound treated cells. The results represent the means of three independent experiments ± the standard deviation. ***, *P*<0.001.
(TIF)

S4 Fig The compound treatment retarded mouse growth. Mice body weights were measured before and after the treatment for 7, 14 and 21 days. The results represent the means of body weights of five mice ± the standard deviation. *, *p*<0.05.
(TIF)

S5 Fig PRMT5 inhibitors selectively affect PRMT5-target genes in lung cancer cells. RNAs were isolated from cells cultured in the presence of DMSO, EPZ015666 (Sigma-Aldrich) (50 μM), or C9a (50 μM) for 24 hrs and submitted to RT-RCR analysis of gene expression in cancer cell lines. Relative mRNA levels = mRNA in cells treated with PRMT5-inhibtor/mRNA in DMSo-treated cells.
(TIF)

S6 Fig The compounds C9 and C9a do not inhibit the cyclin-dependent kinases (CDKs). A, Chemical structures of CDK inhibitors Roscovitine and Polbociclib (Selleckchem.com) and compounds C9 and C9a. B, Western blot of protein extracts (10 μg) derived from A549 cells treated (4 h) with DMSO (lane 1) or 20 μM of compound C9 (lanes 2), C9a (lanes 3), Roscovitine (lanes 4), or Polbociclib (lane 5) with anti-Rb pS811 (Abcam, ab109399), -Rb pT821 (Abcam, ab4787), -Rb (Abcam, ab24) or -actin (Sigma-Aldrich) antibody.
(TIF)

S1 Data Raw data of cell cycle analysis. Cells were treated with compounds and the cell-cycle distributions were determined by flow cytometry analysis (BD Accuri^TM^ C6 Flow Cyometer).
(PDF)

S1 Table Raw data of real-time PCR analysis. RNAs were isolated from cells and submitted for real-time PCR analysis with gene-specific primers as indicated.
(XLSX)
