# Supplementary figures and images for: Selective small-chemical inhibitors of protein arginine methyltransferase 5 with anti-lung cancer activity
Source: PLoS One. 2017 Aug 14;12(8):e0181601. doi: 10.1371/journal.pone.0181601 (PMC5555576; doi:10.1371/journal.pone.0181601)

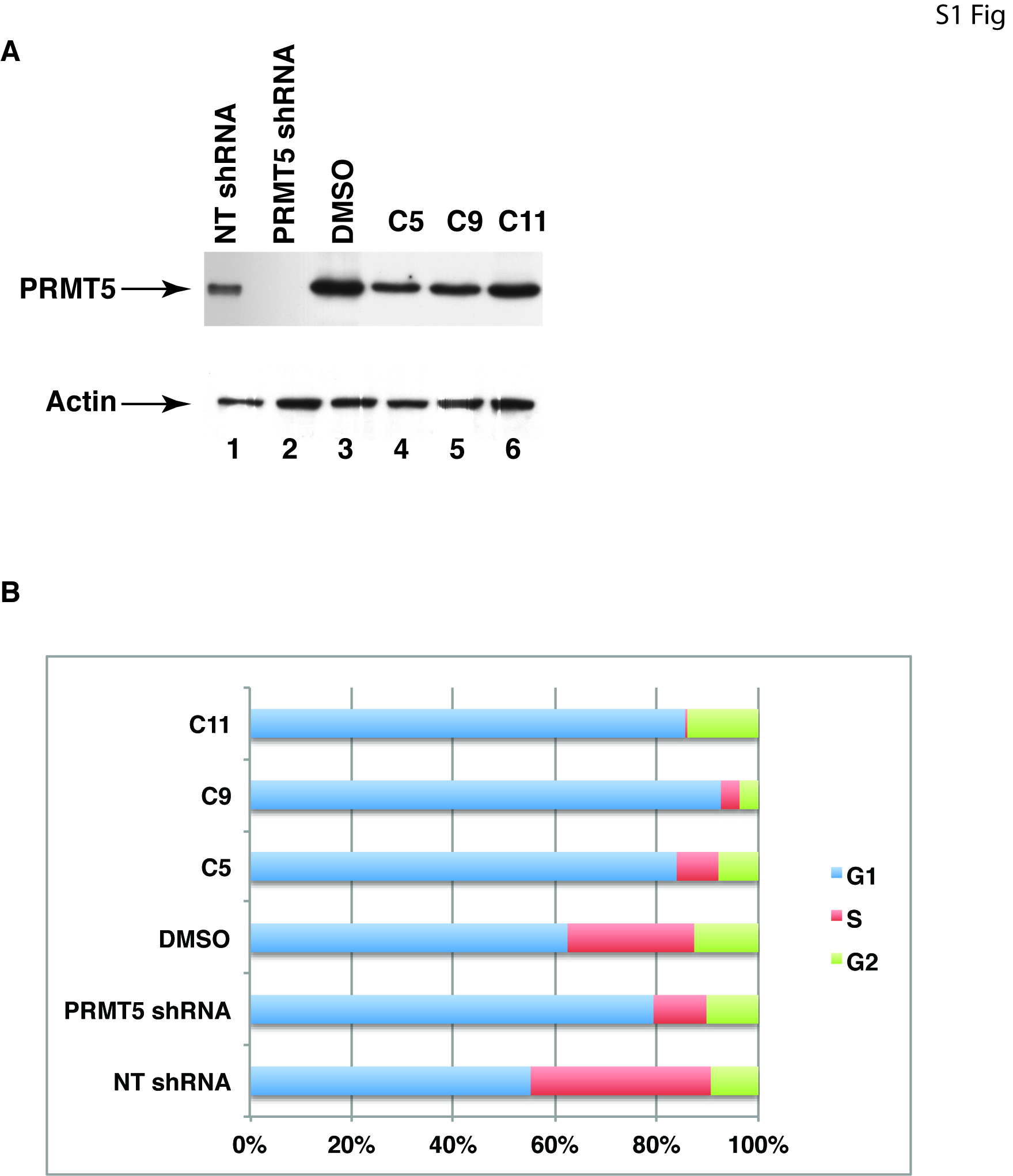

Supplement: S1 Fig — The chemical compound was dissolved in 5% DMSO-0.5% Tween 80. BALB/c mice (8–10 weeks old) were randomized and administrated the vehicle (0.1 ml) or compound C9 dissolved in the vehicle (0.1 ml) by intraperitoneal (I.P.) injection at the dosage of 25, 50 or 100 mg/kg. Mice were sacrificed and blood samples were collected immediately before (0 h, n = 5) and at 2 (n = 5), 4 (n = 5), 6 (n = 5) and 8 (n = 5) h after compound administration. Plasma samples were sent to the Pharmaceutical Development Center at MD Anderson Cancer Center for LC-MS/MS assay to determine compound concentrations. (TIF) [file pone.0181601.s001.tif]

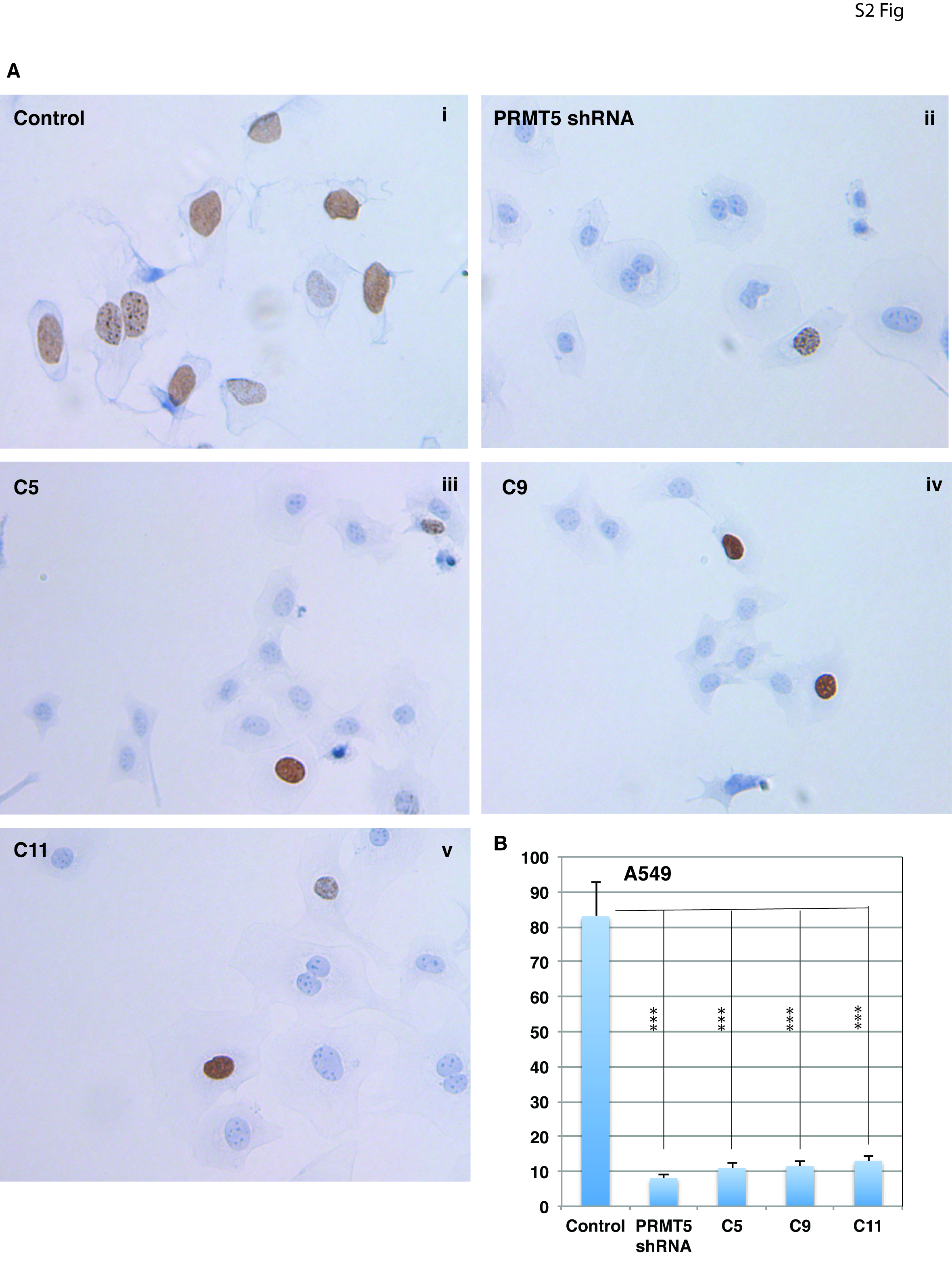

Supplement: S2 Fig — A549 cells were infected with the lentivirus expressing non-target (NT) or PRMT5 shRNA as described previously by us [27]. A549 cells were grown in the presence of DMSO or 20 μM of the compound C5, C9 or C11 for 2 days. A, PRMT5 protein levels in A549 cells. Whole cell lysates were prepared and submitted for Western blot analysis with anti-PRMT5 (top) or anti-actin (bottom) antibody. B, Cell cycle distributions. Cells were harvested, washed with phosphate-buffered saline (PBS) and fixed in 70% ethanol at 4 oC overnight. Cells were collected and stained with propidium iodide (PI). The cell-cycle distributions were determined by flow cytometry analysis (BD AccuriTM C6 Flow Cyometer). (TIF) [file pone.0181601.s002.tif]

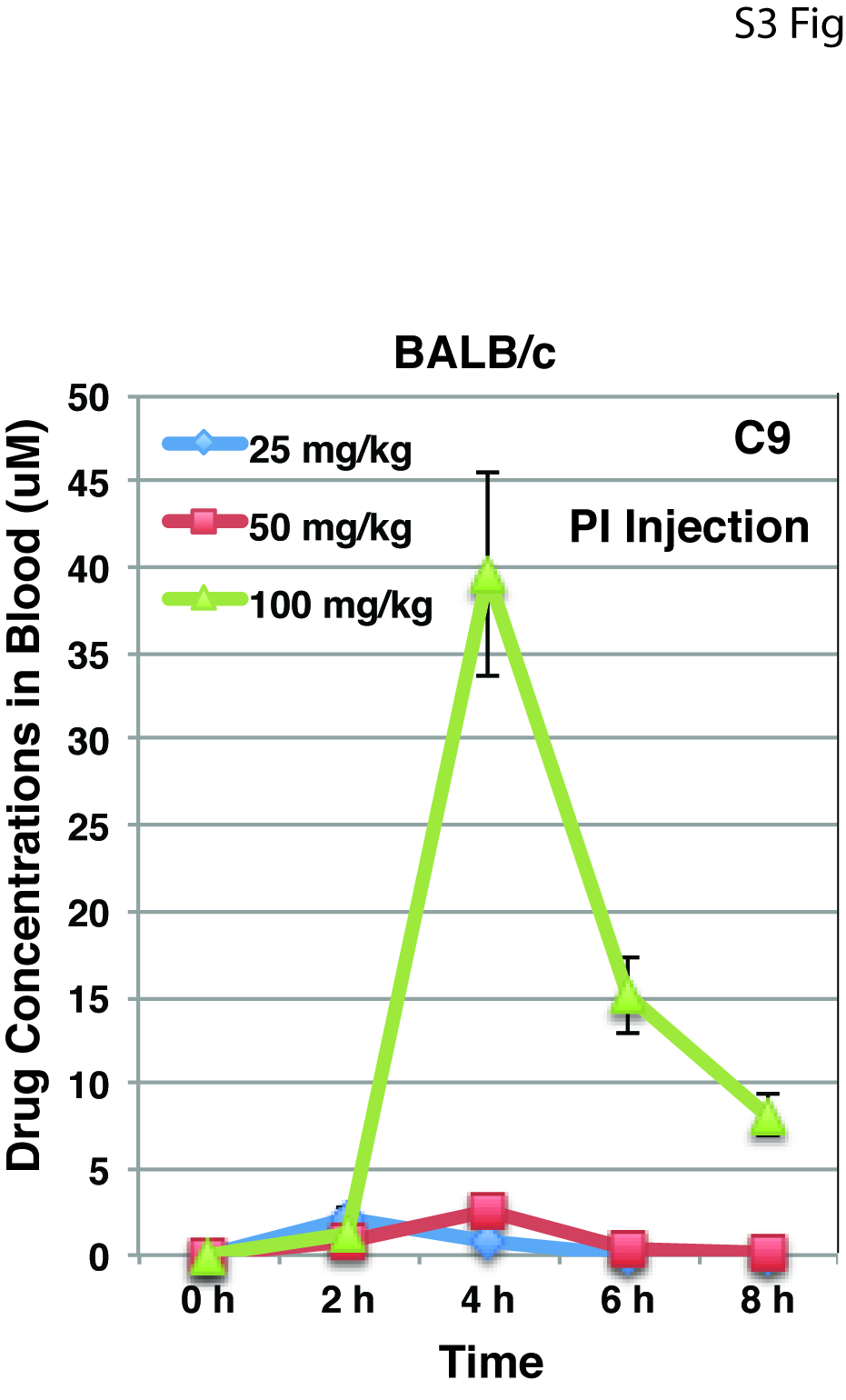

Supplement: S3 Fig — A549 cells were infected with the lentivirus expressing NT or PRMT5 shRNA. A549 cells were grown in the presence of DMSO or compound C5, C9 or C11 (20 μM) for 2 days. A, Bromodeoxyuridine (BrdU) (BD Biosciences) incorporation assay. Cells were plated on a Chamber slide (BD falcon) and cultured in the presence of 10 μM BrdU for 4 h. The BrdU-positive cells (brown) were detected by immunostaining with the monoclonal anti-BrdU antibody (BD Biosciences) and observed under a microscope. B, Percentage of BrdU-positive cells in control, PRMT5-silencing or compound treated cells. The results represent the means of three independent experiments ± the standard deviation. ***, P<0.001. (TIF) [file pone.0181601.s003.tif]

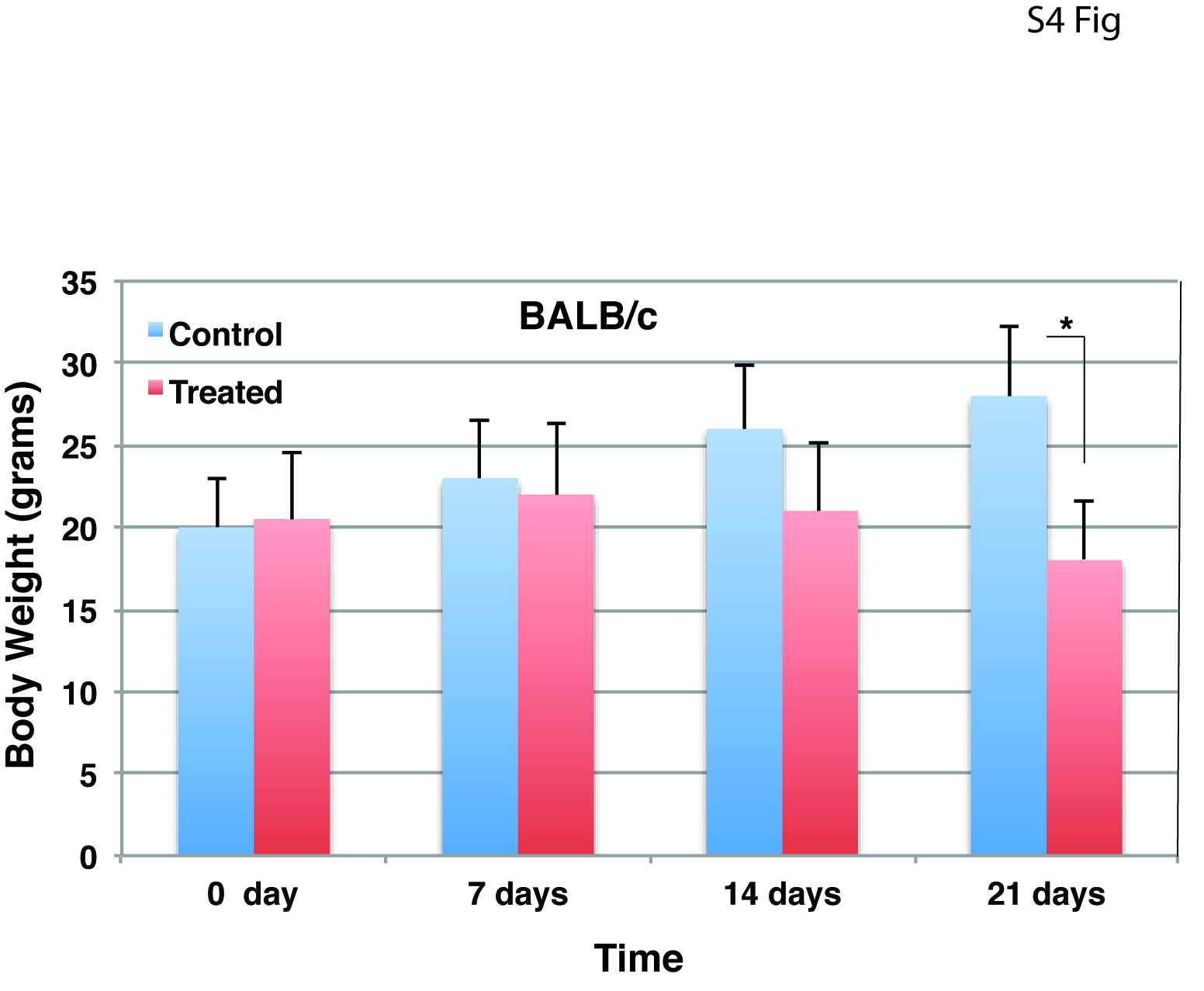

Supplement: S4 Fig — Mice body weights were measured before and after the treatment for 7, 14 and 21 days. The results represent the means of body weights of five mice ± the standard deviation. *, p<0.05. (TIF) [file pone.0181601.s004.tif]

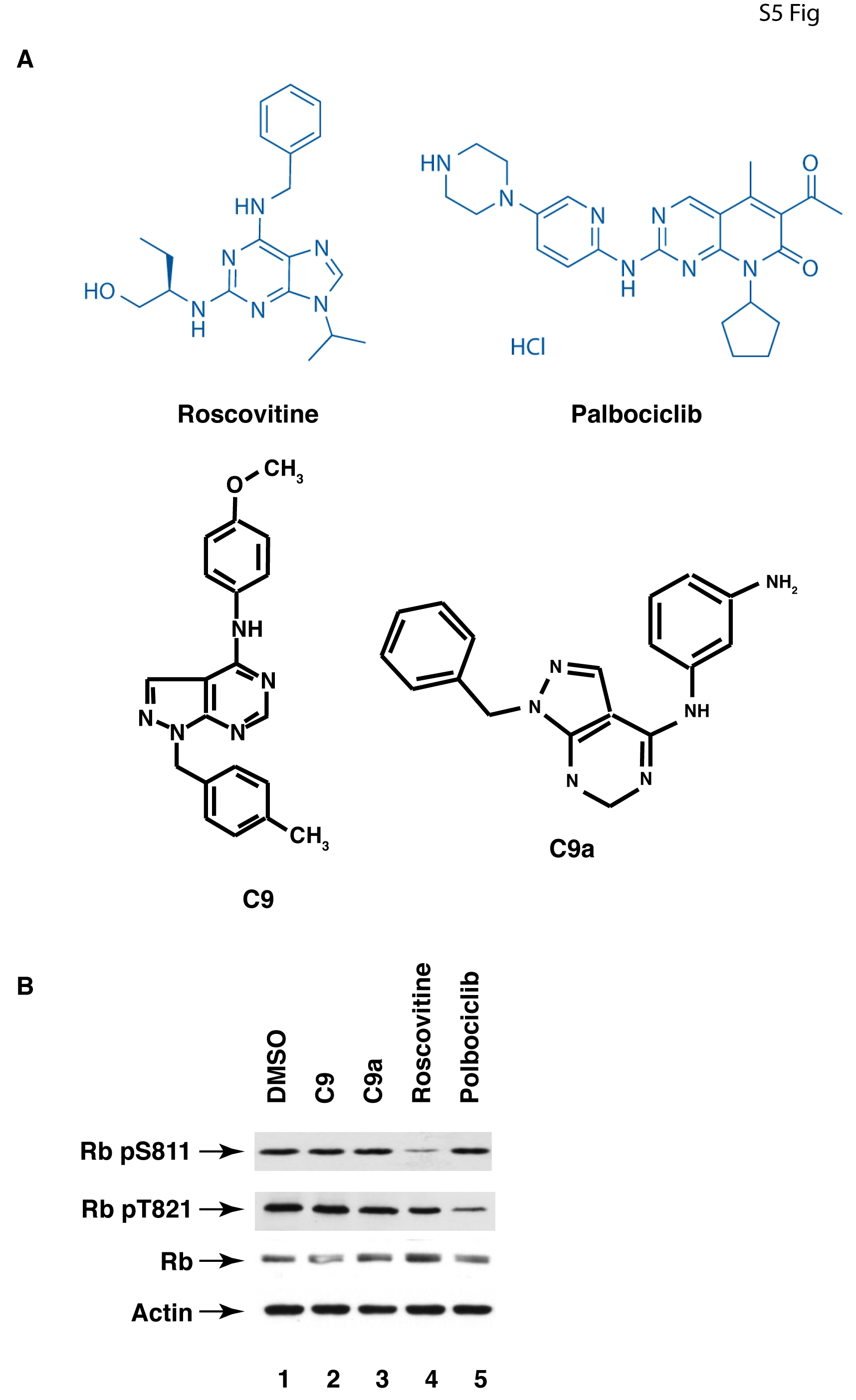

Supplement: S5 Fig — RNAs were isolated from cells cultured in the presence of DMSO, EPZ015666 (Sigma-Aldrich) (50 μM), or C9a (50 μM) for 24 hrs and submitted to RT-RCR analysis of gene expression in cancer cell lines. Relative mRNA levels = mRNA in cells treated with PRMT5-inhibtor/mRNA in DMSo-treated cells. (TIF) [file pone.0181601.s005.tif]

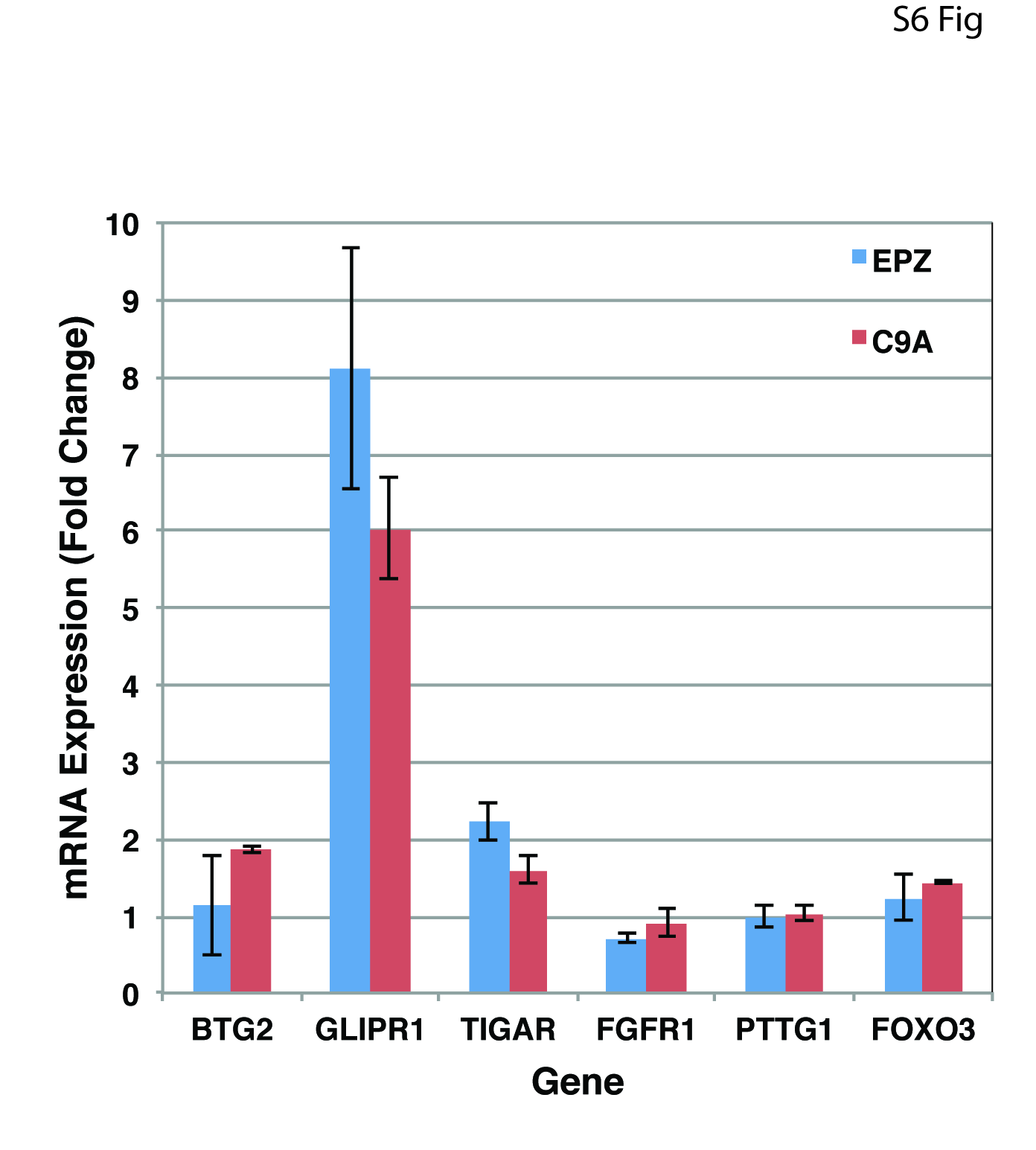

Supplement: S6 Fig — A, Chemical structures of CDK inhibitors Roscovitine and Polbociclib (Selleckchem.com) and compounds C9 and C9a. B, Western blot of protein extracts (10 μg) derived from A549 cells treated (4 h) with DMSO (lane 1) or 20 μM of compound C9 (lanes 2), C9a (lanes 3), Roscovitine (lanes 4), or Polbociclib (lane 5) with anti-Rb pS811 (Abcam, ab109399), -Rb pT821 (Abcam, ab4787), -Rb (Abcam, ab24) or -actin (Sigma-Aldrich) antibody. (TIF) [file pone.0181601.s006.tif]
