# Supplementary material for: Selective small-chemical inhibitors of protein arginine methyltransferase 5 with anti-lung cancer activity
Source: PLoS One. 2017 Aug 14;12(8):e0181601. doi: 10.1371/journal.pone.0181601 (PMC5555576; doi:10.1371/journal.pone.0181601)
Supplement: S1 Data — Cells were treated with compounds and the cell-cycle distributions were determined by flow cytometry analysis (BD AccuriTM C6 Flow Cyometer). (PDF) [file pone.0181601.s007.pdf]

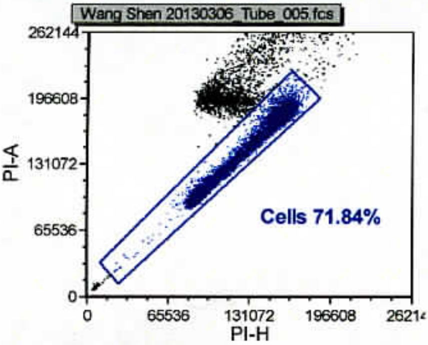

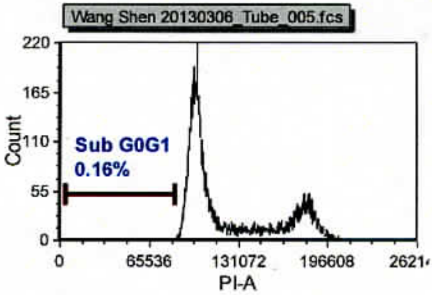

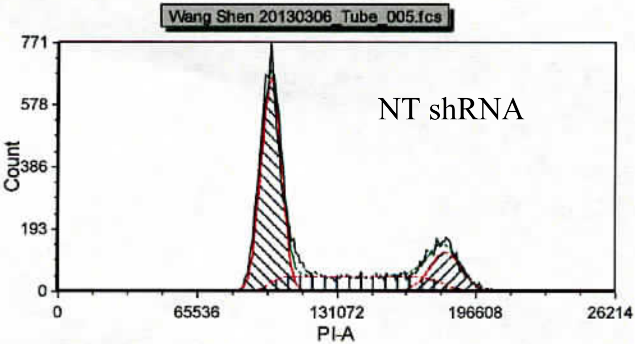

Cycle	G1 Mean	G1 CV	%G1	G2 Mean	G2 CV	%G2	%S	G2/G1	%Total	B.A.D.
Diploid	100378.11	4.73	55.35	182799.16	4.16	16.41	28.25	1.82	100.00	0.00

Interpretation

MultiCycle suggestions (a guideline only):
No abnormal DNA content is observed.
The diploid %S=28.2, %G2=16.4
The S Phase confidence is fair
Note: inter-model error,

Model	Dip %G2	Dip %S	Chis
SL S0	16.41	28.25	2.99
SL CL S0	15.87	28.55	2.64
+G2/G1 Fixed	0.00	42.39	8.09
+Aggregates	0.66	41.22	8.89
+S Order = 1	19.14	27.38	2.58
+CVs Fixed	19.35	27.27	2.56

Experiment Statistics

Chi sq: 2.99
BAD: 0.00
Number of cells: 14368.00
Number of cycles: 1.00
Cycle fit model: 1 Cycle

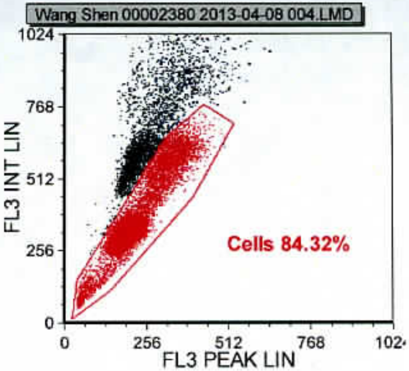

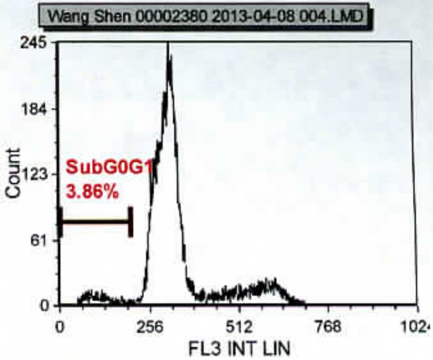

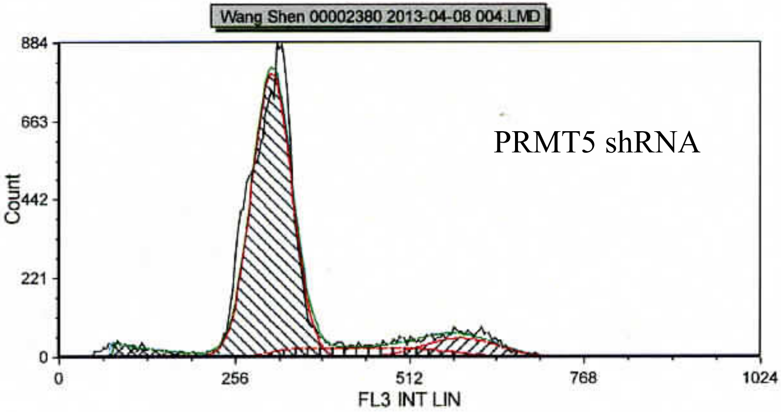

Cycle	G1 Mean	G1 CV	%G1	G2 Mean	G2 CV	%G2	%S	G2/G1	%Total	B.A.D.
Diploid	310.37	9.41	79.40	589.11	9.42	10.20	10.40	1.90	100.00	0.61

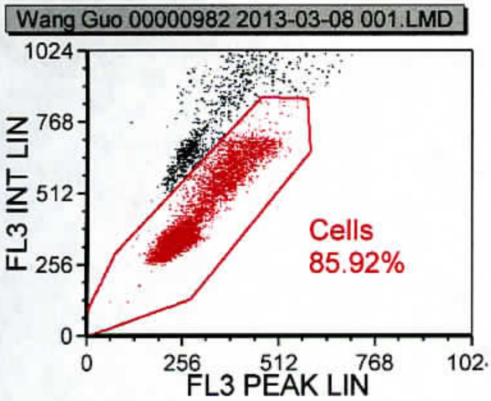

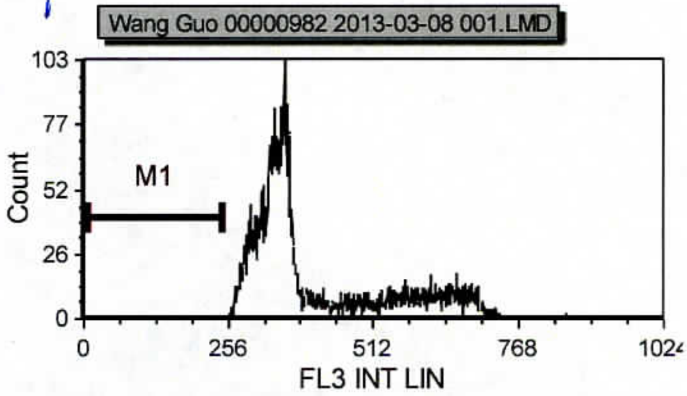

Parameter	# of Events	% of gated cells	Median	Geometric Mean	CV	Peak Value	Peak Channel
FL3 INT LIN	4	0.05	147.50	153.81	34.18	1.00	116.00

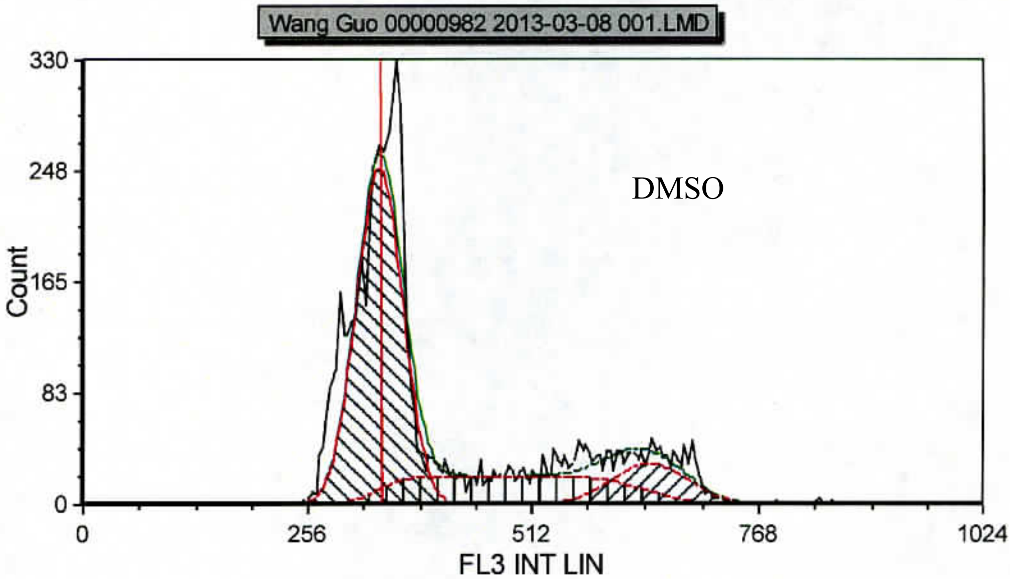

Cycle	G1 Mean	G1 CV	%G1	G2 Mean	G2 CV	%G2	%S	G2/G1	%Total	B.A.D.
Diploid	337.19	7.82	62.49	647.93	6.76	12.60	24.91	1.92	100.00	0.00

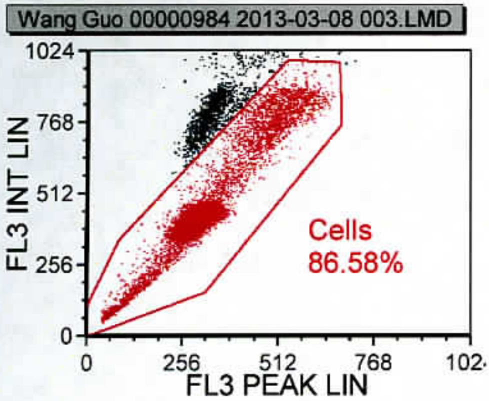

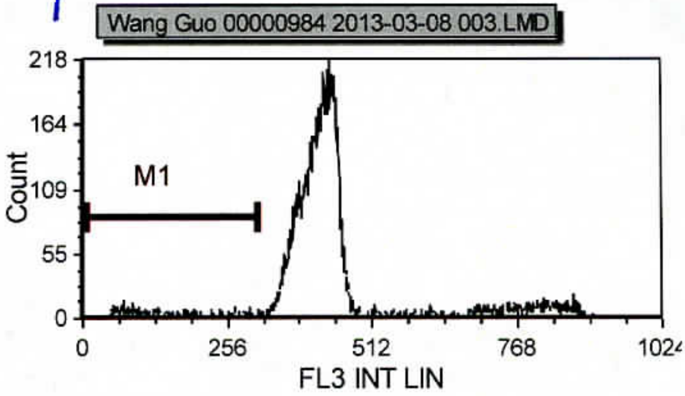

Parameter	# of Events	% of gated cells	Median	Geometric Mean	CV	Peak Value	Peak Channel
FL3 INT LIN	1128	6.20	147.50	138.55	49.61	14.00	72.00

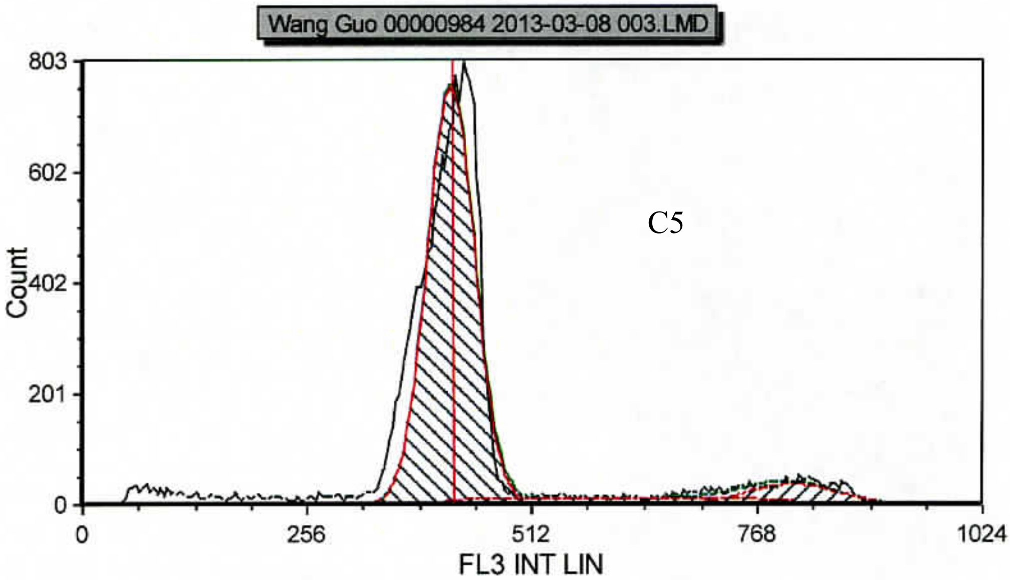

Cycle	G1 Mean	G1 CV	%G1	G2 Mean	G2 CV	%G2	%S	G2/G1	%Total	B.A.D.
Diploid	419.65	6.44	83.93	812.35	6.23	7.85	8.22	1.94	100.00	0.00

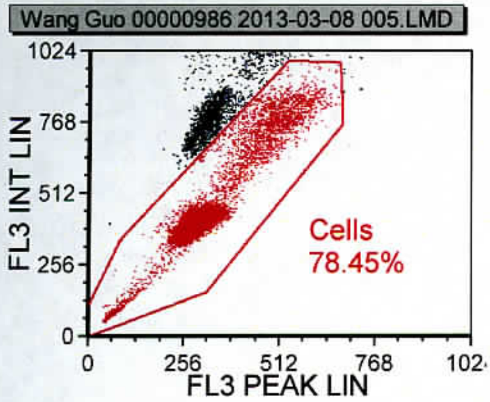

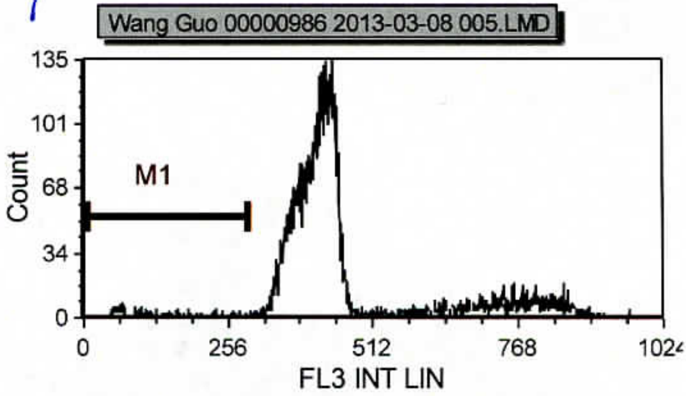

Parameter	# of Events	% of gated cells	Median	Geometric Mean	CV	Peak Value	Peak Channel
FL3 INT LIN	425	3.58	102.00	109.82	53.70	8.00	69.00

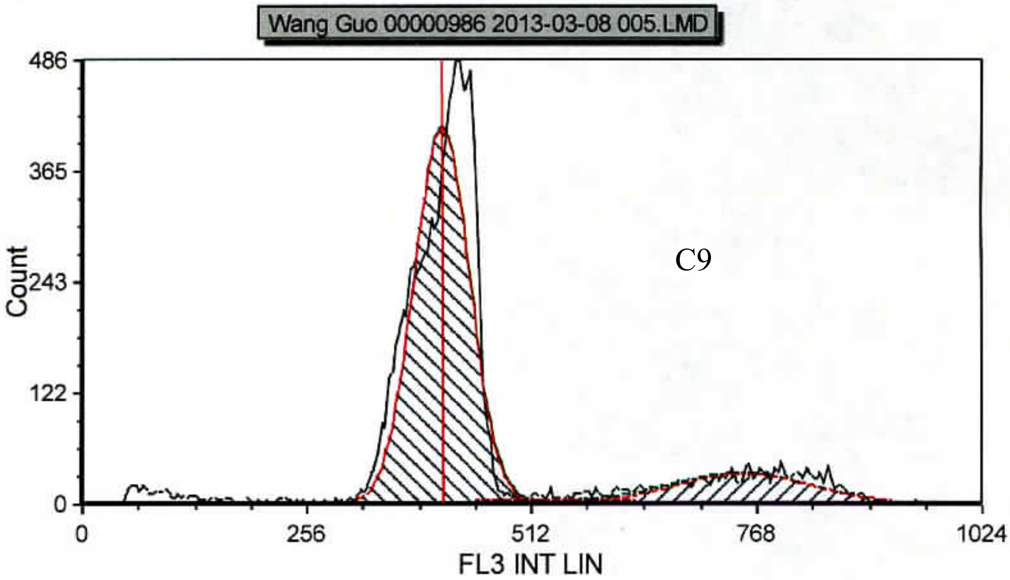

C9

Cycle	G1 Mean	G1 CV	%G1	G2 Mean	G2 CV	%G2	%S	G2/G1	%Total	B.A.D.
Diploid	409.13	7.78	80.63	757.18	10.52	16.17	3.20	1.85	100.00	0.00

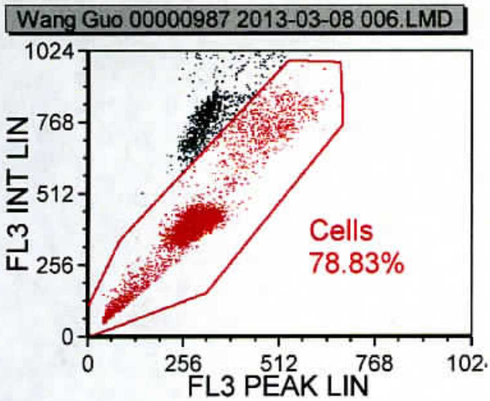

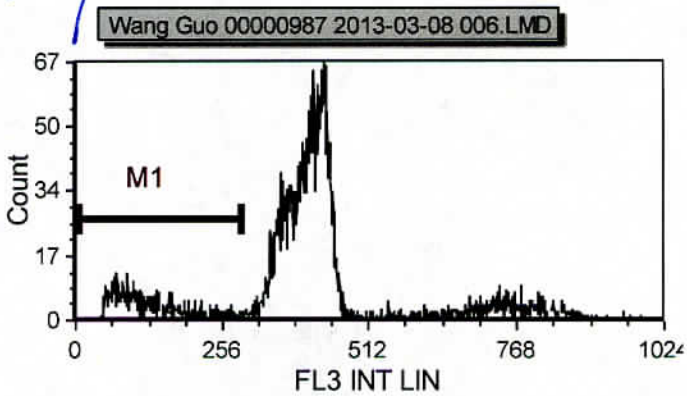

Parameter	# of Events	% of gated cells	Median	Geometric Mean	CV	Peak Value	Peak Channel
FL3 INT LIN	848	13.73	110.00	114.28	49.26	12.00	70.00

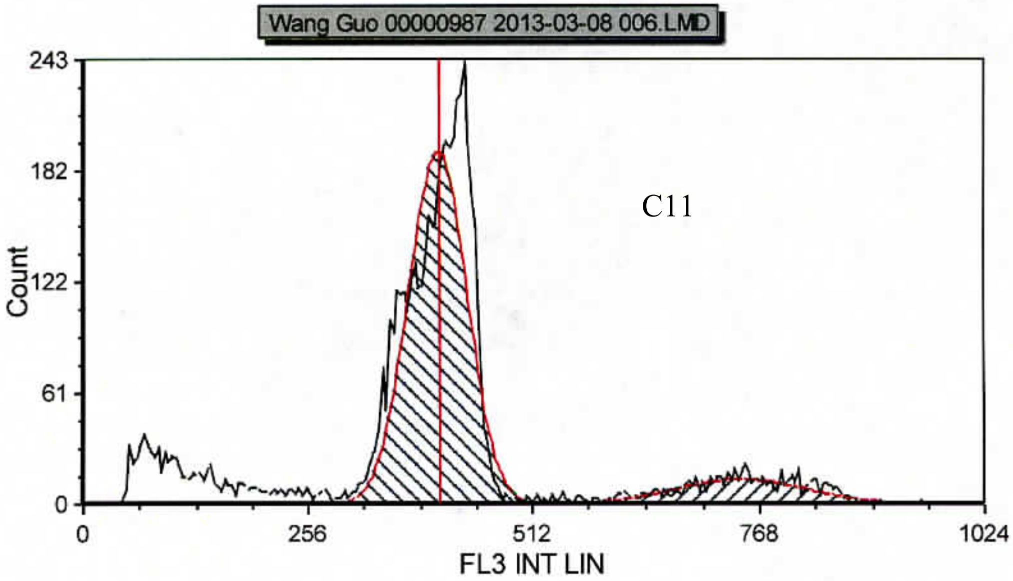

Cycle	G1 Mean	G1 CV	%G1	G2 Mean	G2 CV	%G2	%S	G2/G1	%Total	B.A.D.
Diploid	403.11	8.24	85.61	751.09	10.03	13.96	0.43	1.86	100.00	0.00
